# PAQR3 Inhibits Non-small Cell Lung Cancer Growth by Regulating the NF-κB/p53/Bax Axis

**DOI:** 10.3389/fcell.2020.581919

**Published:** 2020-10-06

**Authors:** Qiang Guo, Xi-Xian Ke, Shi-Xu Fang, Wei-Long Gao, Yong-Xiang Song, Cheng Chen, Hong-Ling Lu, Gang Xu

**Affiliations:** ^1^Department of Thoracic Surgery, The Affiliated Hospital of Zunyi Medical University, Zunyi, China; ^2^Department of Cardiac Surgery, The Affiliated Hospital of Guizhou Medical University, Guiyang, China; ^3^Department of Biochemistry, Zunyi Medical University, Zunyi, China

**Keywords:** non-small cell lung cancer, PAQR3, prognosis, GSEA, tumor growth

## Abstract

**Background:**

The expression of progestin and adipoQ receptor 3 (PAQR3) is generally downregulated in multiple tumors, which is associated with tumor progression and poor prognosis.

**Methods:**

The clinical value of PAQR3 was analyzed using various databases and in 60 patients with non-small cell lung cancer (NSCLC). In addition, cell counting kit-8 (CCK-8), colony formation, and flow cytometry assays were used to evaluate the effect of PAQR3 on the growth of NSCLC cells *in vitro*. Gene set enrichment analysis (GSEA) was performed to investigate the possible mechanism through which PAQR3 is involved in the progression of lung cancer. Furthermore, western blotting was employed to verify the relevant mechanism.

**Results:**

The expression of PAQR3 was decreased in 60 NSCLC patients and was related to the histological subtype, lymph node metastasis, tumor size, and diagnosis of NSCLC. Patients with lung adenocarcinoma with increased PAQR3 expression tended to have a better prognosis. Besides, PAQR3 inhibited proliferation, clone formation, and cycle transition in NSCLC cells, but induced apoptosis. The results of GSEA showed that PAQR3 regulated the progression of lung cancer by affecting cell cycle, DNA replication, and the p53 signaling pathway. We confirmed that PAQR3 overexpression inhibited the expression of NF-κB, while it increased the expression of p53, phospho-p53, and Bax. On the contrary, PAQR3 inhibition played an opposite role in these proteins.

**Conclusion:**

PAQR3 inhibited the growth of NSCLC cells through the NF-κB/P53/Bax signaling pathway and might be a new target for diagnosis and treatment.

## Introduction

Non-small cell lung cancer (NSCLC) is the commonest type of lung cancer and accounts for approximately 85% of lung cancer cases (Florczuk et al., 2017; Dai et al., 2018; Siegel et al., 2020). Its common tissue subtypes include adenocarcinoma, squamous cell carcinoma, and large cell carcinoma. Lung cancer is not only the main cause of death among men with tumors, but also a common disease associated with cancer-related deaths among female patients (Siegel et al., 2020). The increasing awareness of cancer and improvements in diagnostic and treatment techniques, such as surgery, chemotherapy, and radiotherapy, have provided some survival benefits to NSCLC patients, although the 5-year overall survival (OS) remains low. In recent years, the emergence of targeted therapy has resulted in revolutionary changes in the treatment of lung cancer. For instance, targeted treatment of NSCLC patients with *EGFR* mutations could improve their survival time (Ghafoor et al., 2018; Hsu et al., 2019), and targeted therapy for anaplastic lymphoma kinase positive disease could also improve the prognosis of patients with metastatic NSCLC (McCoach et al., 2017). However, the efficacy is far from satisfactory. Therefore, it is essential to explore new therapeutic targets to improve the prognosis of NSCLC patients.

Progestin and adipoQ receptor 3 (PAQR3) is a newly discovered tumor suppressor (Lei et al., 2020). The expression level of PAQR3 in colon cancer tissues is significantly lower than that in normal colon tissues, and this change is closely associated with the malignant degree of colon cancer (Wang et al., 2012). In human breast cancer, the expression of PAQR3 is decreased and negatively correlated with that of HER2. PAQR3 inhibits the proliferation, clone formation, migration, and invasion of breast cancer cells (Li et al., 2015). The expression of PAQR3 is decreased and closely associated with serum alpha-fetoprotein (AFP), clinical stage, tumor size, and survival time in hepatocellular carcinoma. Consistently, it inhibits tumor proliferation and migration (Wu et al., 2014). In addition, the expression of PAQR3 in esophageal cancer tissues is lower than that in normal esophageal tissues, and is significantly correlated with tumor size, lymph node metastasis, and local recurrence among esophageal cancer patients. In addition, it inhibits the proliferation, cell cycle, clone formation, migration, and invasion of esophageal cancer cells, as confirmed in a nude mouse xenograft model (Zhou et al., 2017; Bai et al., 2017, 2018). In addition, PAQR3 expression is decreased in some other tumors such as osteosarcoma, gastric cancer, and breast cancer, and the functions in these tumor tissues are consistent with those mentioned above, and include the inhibition of the growth and migration of tumor cells (Ling et al., 2014; Li et al., 2015; Ma et al., 2015; Zhao et al., 2017). In summary, *PAQR3* played a role in the occurrence and development of all reported cancers as a tumor suppressor gene, and no opposite effect has been found.

At present, PAQR3 expression has been found to be decreased in lung cancer tissues and is significantly correlated with the pathological classification, degree of differentiation, TNM stage, and lymph node metastasis in patients with lung cancer. Kaplan–Meier survival analysis showed that the positive expression rate of PAQR3 protein was positively associated with survival time and the 5-year survival rate (Liang et al., 2017). Li et al. (2018) found that PAQR3 inhibits cell proliferation and cell cycle transition, while promoting apoptosis in NSCLC cells through the PI3K/AKT signaling pathway. The Cancer Genome Atlas (TCGA) and Gene Expression Omnibus (GEO) databases contain a large number of high-throughput sequencing results, while Oncomine, Timer, and Ualcan database results are derived from TCGA and GEO data. We used the database in advance to demonstrate that PAQR3 expression is increased in NSCLC tissues. This is contrary to the results reported by Liang et al. (2017). In addition, the clinical value and biological function of PAQR3 in NSCLC have not been fully elucidated, particularly in regulating the occurrence and development of NSCLC. Therefore, in the present study, we analyzed the expression of PAQR3 and its clinical value in NSCLC, and explored its function in the progression of NSCLC to provide a new target for molecular diagnosis and treatment of lung cancer.

## Materials and Methods

### Oncomine, Timer, and Ualcan Databases

The screening criteria for the Oncomine database^1^ were: (1) gene: *PAQR3*; (2) analysis type: cancer vs. normal analysis; (3) data type: mRNA; (4) *p* < 0.01; and (5) foldchange: 1.5. The expression of PAQR3 in different types of cancer was analyzed in the gene expression module of the Timer database^2^. Moreover, its expression in NSCLC and normal samples was analyzed in the Ualcan database^3^, and the correlations between the expression level and clinicopathological features (race, weight, smoking history, cancer stage, grade, etc.) of NSCLC patients were analyzed. The clinical data are detailed in Supplementary Table S1.

### PrognoScan and Kaplan-Meier Plotter Databases

The data in the PrognoScan database^4^ mainly originate from the RNA data in the GEO database. The data in the Kaplan–Meier Plotter database^5^ comes from the RNA data of the TCGA and GEO databases. Using the PrognoScan database, we analyzed the relationship between the expression level of PAQR3 and the prognosis of patients. The screening criterion was COX *p* < 0.05 which means statistically significant. For the Kaplan-Meier Plotter database, we used the median value of PAQR3 expression as the grouping standard to analyze the relationship between PAQR3 expression level and the prognoses of lung adenocarcinoma (LUAD) and lung squamous carcinoma (LUSC) patients.

### Analysis of Biological Functions Related to PAQR3

The Cancer Cell Line Encyclopedia (CCLE) database contains more than 1000 databases of human cancer cells. In the present study, PAQR3 co-expressed genes were screened using the screening criteria of *r* (*r* > 0.4 or < −0.4) and *p* < 0.001 through the mRNA expression data of lung cancer cells downloaded from the CCLE database, and analysis of Gene Ontology (GO), analysis of the Kyoto Encyclopedia of Genes and Genomes (KEGG), and gene set enrichment analysis (GSEA) were performed. GO enrichment and KEGG pathway analyses were performed using the clusterProfiler software package on the R platform. GSEA is a method used to reveal mRNA level data via basic knowledge (Zhou et al., 2020). Gene expression data from the CCLE were divided into high and low expression groups according to the median level of PAQR3 mRNA expression, and GSEA was used to explore the influence of different levels of PAQR3 on each gene and to analyze the mechanism underlying the involvement of PAQR3 in the progression of lung cancer. The genome was sequenced 1000 times per analysis. In addition, the level of PAQR3 was used as a phenotypic marker. The nominal *p*-value (NOM p) and the normalized enrichment score were used to classify enrichment pathways in each phenotype (Guo et al., 2020).

### Clinical Samples

Cancerous tissues and normal tissues located 5 cm adjacent to cancerous tissues were collected from 60 NSCLC patients who underwent surgery at the Department of Thoracic Surgery of Zunyi Medical University from December 2017 to January 2019. None of the patients was treated with neoadjuvant chemotherapy, radiotherapy, immunosuppressant therapy, biotherapy, or targeted therapy before surgery. Their clinical data are shown in Supplementary Table S2. The study protocol was approved by the Ethics Committee of The Affiliated Hospital of Zunyi Medical University (approval no. KLLY-2018-095). All 60 patients provided written informed consent prior to undergoing surgery.

### Cell Culture

A549 and H1299 NSCLC cells were purchased from the cell bank of the typical Culture Preservation Committee of the Chinese Academy of Sciences. RPMI-1640 complete culture medium was used and incubated routinely in 5% CO_2_ at 37°C.

### Quantitative Reverse Transcription Polymerase Chain Reaction

Total RNA was extracted from NSCLC tissues according to the manufacturer’s instructions (Invitrogen, United States). cDNA was synthesized using a reverse transcription kit (Takara, Japan). The expression of PAQR3 mRNA was detected by RT-qPCR. Glyceraldehyde 3-phosphate dehydrogenase (GAPDH) was used as an internal control. The primer sequences were: PAQR3: 5′-TGTCGAAGATGGATGGCATTAGA-3′ (forward), 5′-ACCTGACGCCAGTAGTATTACACACA-3′ (reverse) and GAPDH: 5′-AACGGATTTGGTCGTATTG-3′ (forward), 5′-GGAAGATGGTG ATGGGATT-3′ (reverse). These experiments were performed in triplicate.

### Western Blot Assay

Total proteins were extracted from tissues and cells and quantified using the bicinchoninic acid method according to the manufacturer’s instructions (Solarbio, China) and separated by electrophoresis. Extracted proteins were then resolved via 10% sodium dodecyl sulfate-polyacrylamide gel electrophoresis (SDS-PAGE) and electro-transferred onto a polyvinylidene difluoride (PVDF) membrane. The membranes were blocked with 5% skimmed milk on a shaker for 1 h. The primary antibodies used in the present study were 1:300 anti-PAQR3 (Abcam, Cambridge, United Kingdom), 1:1000 anti-NF-κB (HuaBio, China), 1:2000 anti-p53, 1:3000 anti-Bax (Proteintech, China), and 1:1000 anti-p-p53 (CST, United States). A 1:3000 anti-GAPDH (Bioss, China) and 1: 2000 anti-β-tubulin (HuaBio, China) were used as controls. Images were obtained using Image Lab^TM^ Software (ChemiDoc^TM^ XRS+, Bio-Rad Laboratories Inc., Hercules, CA, United States). These experiments were performed in triplicate.

### Generation of Stable Cells

The cDNA sequence of PAQR3 (NM_001040202.2) was obtained from NCBI. Lentiviral-based Lv201CT and psi-LVRU6GP vector (containing puromycin and enhanced green fluorescent protein) were constructed by GeneCopoeia, Inc (Rockville, MD, United States). Lentiviral ORF cDNA clones for PAQR3 (EX-E2253-Lv201 with N-eGFP) and empty vector control plasmid (EX-NEG-Lv201) were obtained from GeneCopoeia. Specifically, an shRNA with a target sequence (LPP-HSH003695-LVRU6GP) in the PAQR3 coding DNA sequence and one non-targeting shRNA as negative control (LPP-CSHCTR001-LVRU6GP) were used in this experiment (referred to as Si-PAQR3 and Si-NC, respectively). The sequence of the interference control group was ACAGAAGCGATTGTTGATC, and the sequence of the interference PAQR3 group was GCATTAGATTATGCAGGAATT. The stable overexpression or knockdown model of PAQR3 in NSCLC cells (A549 and H1299 cells) was performed using an EndoFectin transfection (GeneCopoeia, Inc.) following the manufacturer’s protocol. Stable NSCLC cells were selected in a medium containing puromycin (Solaibo, Beijing, China), 2 μmol/L of A549, and 4 μmol/L of H1299 for 14 days. After 2–3 passages in the presence of puromycin, the cultured cells were evaluated to verify that the PAQR3 overexpression or knockdown model was constructed successfully and used for experiments.

### Cell Counting Kit-8 Assay

The cell counting kit-8 (CCK-8) assay was used to evaluate the effect of PAQR3 on the proliferation of A549 and H1299 cells. The A549 and H1299 cells in the logarithmic growth phase from the overexpression control group (Vector) and PAQR3 overexpression group (PAQR3-OV), or from the knockdown control group (Si-NC) and PAQR3 knockdown group (Si-PAQR3) were seeded in 96-well culture plates. The number of cells was 2.5 × 10^3^ cells/well, and wells were set up in multiples of two. According to the operation of the kit, 10 μl/hole of CCK-8 was added and incubated at 37°C and with 5% CO_2_ for 3 h. The optical density was measured at 450 nm using a microplate reader (Bio-Rad, United States). These experiments were performed in triplicate.

### Clone Formation Assay

Stable cells in the logarithmic growth phase were seeded in a 6-well culture plate containing medium at 1500 cells/well. The cells were grouped as described above and incubated at 37°C for 2 to 3 weeks. The culture was terminated at the right time, fixed with 4% paraformaldehyde (Solaibo, Beijing, China) for 10 min, stained with crystal violet (Sigma-Aldrich, China) for 30 min, and colonies containing more than 10 cells were observed under a microscope. These experiments were performed in triplicate.

### Cell Cycle and Apoptosis

Stable A549 and H1299 cells (4 × 10^5^ cells/well) were plated in cell culture dishes (60 mm × 60 mm). For the cell cycle analysis, cells were collected by trypsinization after 2 days, washed once with phosphate-buffered saline (PBS), and analyzed according to the instructions stipulated in the cell cycle kit (ABP Biosciences, China). For cell apoptosis analysis, cells were collected after transfection by trypsinization (without ethylenediaminetetraacetic acid), washed once with PBS, added to 500 μL of 1 × binding buffer, and stained with 1 μL of Annexin V-PE and 5 μL of 7-AAD (Nanjing Kaiji, China). Flow cytometry was used for subsequent detection. These experiments were performed in triplicate.

### Statistical Analysis

The differences in expression between cancerous and normal tissues were calculated using the chi-squared test. Cell proliferation, number of clones, cycle, and apoptosis were analyzed using the Student *t*-test or one-way analysis of variance. We performed receiver operating characteristic (ROC) curve analysis on PAQR3 mRNA expression in normal and cancer tissues to evaluate whether PAQR3 has a diagnostic reference value for NSCLC (Wang et al., 2018). In this evaluation, the comprehensive statistic of the area under the curve (AUC) was used for quantitative analysis. The closer the AUC was to 1, the better was the diagnostic effect. All statistical analyses were performed using SPSS version 25.0 (IBM Corp., Armonk, NY, United States), and differences were considered statistically significant at *P* < 0.05. The data are representative of three independent experiments and presented as the mean ± standard deviation. The data were plotted using GraphPad Prism 5.0 (GraphPad Software, San Diego, CA, United States).

## Results

### PAQR3 Was Abnormally Expressed in NSCLC Tissues

**FIGURE 1:**
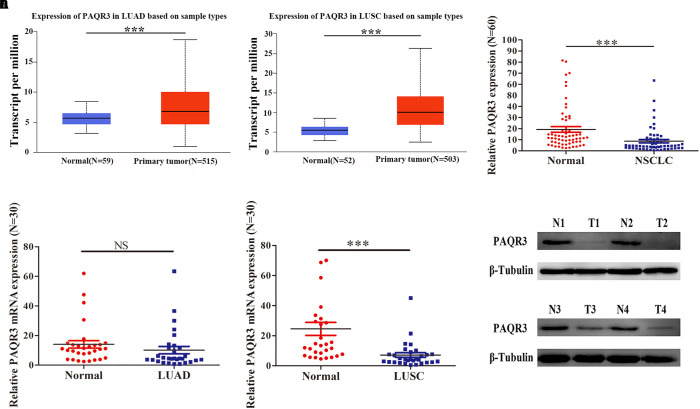
Expression of PAQR3 in Ualcan database and 60 clinical NSCLC samples. **(A,B)** PAQR3 mRNA level in LUAD and LUSC from Ualcan Database. **(C–F)** Expression of PAQR3 in 60 collected NSCLC samples. Note: Normal, normal lung tissue; NSCLC, non-small cell lung cancer; LUAD, lung adenocarcinoma; LUSC, lung squamous carcinoma; ***P < 0.001; NS, no statistical significance; vs. Normal.

In the Oncomine and Timer databases, we found abnormal expression of PAQR3 in various tumors. It was increased in gastric cancer, head and neck tumors, lung cancer, and some other tumors, while it was decreased in breast cancer, melanoma, prostate cancer, and other tumors (Supplementary Figure S1A). Similar results were found in the Timer database (Supplementary Figure S1B). However, to date, numerous studies have reported that PAQR3 is a newly discovered tumor suppressor. Therefore, the expression of PAQR3 mRNA and protein was further verified in the Ualcan database and clinical NSCLC samples. In the Ualcan database, we found that the expression of PAQR3 was increased in LUAD and LUSC (Figures 1A,B). Among 60 collected NSCLC samples, the expression of PAQR3 mRNA in most cancer tissues was decreased significantly (Figure 1C). The expression of PAQR3 mRNA was decreased, while the difference was not significant in the 30 LUAD samples (Figure 1D). In addition, for the other 30 LUSC samples, it was also significantly decreased (Figure 1E). In addition, compared with the paired normal tissues, the expression of PAQR3 protein in 75% (45 cases) of samples was significantly lower (Figure 1F). The quantization diagram is presented in the Supplementary Figure S2.

### PAQR3 Expression Was Correlated With Histological Subtype, Lymph Node Metastasis, and Tumor Size Among NSCLC Patients

**FIGURE 2:**
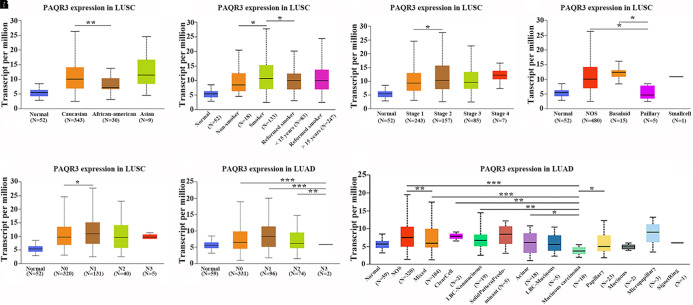
Abnormal expression of PAQR3 mRNA according to the clinicopathological features of NSCLC patients in the Ualcan database. **(A–E)** The clinicopathological features of LUSC patients were race, smoking, clinical stage, histological subtype, and lymph node metastasis, respectively. **(F,G)** Lymph node metastasis and histological subtypes of LUAD patients. NSCLC, non-small cell lung cancer; LUAD, lung adenocarcinoma; LUSC, lung squamous carcinoma; *P < 0.05; **P < 0.01; ***P < 0.001.

In the Ualcan database, we found that the level of PAQR3 mRNA was significantly associated with race, smoking history, clinical stage, histological subtype, and lymph node metastasis among LUSC patients (Figures 2A–E). Among LUAD patients, it was significantly associated with histological subtype and lymph node metastasis (Figures 2F,G). However, in the 60 clinical samples, the level of PAQR3 mRNA was only significantly correlated with tumor size (*P* < 0.05) (Table 1).

**TABLE 1:** The expression of PAQR3 according to clinicopathological features among 60 NSCLC patients.

Clinicopathological parameters		*N*	PAQR3 expression (2-^Δ *Cq*^)	
			Mean ± SD *P*-value	
Gender	Male	28	7.201.70	0.350
	Female	32	9.952.29	
Age	≦60	33	9.172.13	0.705
	>60	27	8.051.95	
Smoking	NO	40	8.711.73	0.964
	YES	20	8.572.44	
Type	LUSC	30	7.171.57	0.308
	LUAD	30	10.162.45	
Tumor size (cm)	≤5	28	11.942.79	0.046
	>5	32	5.801.02	
TNM	I-II	47	9.741.78	0.161
	III	13	4.781.62	
Lymphatic metastasis	NO	43	8.221.91	0.634
	YES	17	9.781.79	
T Stage	T1-T2	45	9.431.83	0.370
	T3-T4	15	6.391.87	

### PAQR3 Is a Potential Diagnostic and Prognostic Marker of NSCLC

**FIGURE 3:**
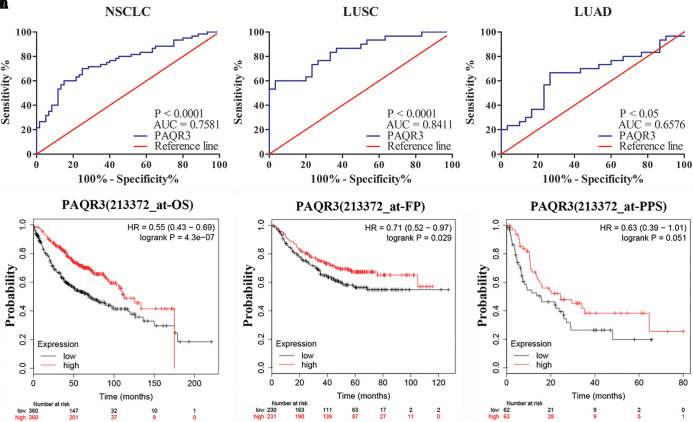
PAQR3 was a potential diagnostic and prognostic marker in NSCLC. **(A)** NSCLC; **(B)** LUAD; **(C)** LUSC; **(D)** OS; **(E)** FP; **(F)** PPS. NSCLC, non-small cell lung cancer; LUAD, lung adenocarcinoma; LUSC, lung squamous carcinoma; AUC, area under the curve; OS, overall survival; FP, first progression; PPS, post-progression survival.

The role of PAQR3 in the diagnosis and prognosis of NSCLC patients was further analyzed. In 60 NSCLC tissues, ROC analysis revealed that the level of PAQR3 was predictive of NSCLC (Figures 3A–C). In the PrognoScan database, the GSE31210 dataset revealed that LUAD patients with elevated PAQR3 levels tended to have a better prognosis (Cox *P* < 0.05) (Supplementary Figure S3). Moreover, the results of the Kaplan–Meier Plotter database were similar to those of the PrognoScan database (Figures 3D–F). Increased PAQR3 expression also predicted better prognosis in LUSC patients, although the difference was not significant.

### PAQR3 Inhibits the Growth of NSCLC Cells

**FIGURE 4:**
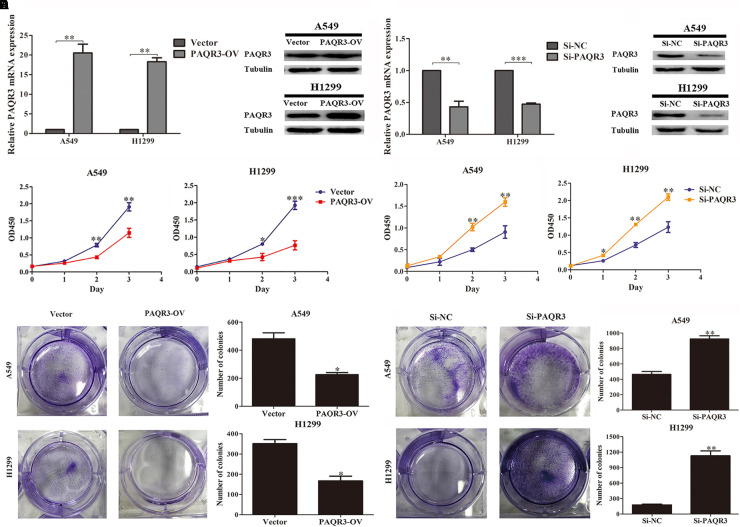
PAQR3 inhibited the proliferation of NSCLC cells. **(A,B)** The levels of PAQR3 mRNA and protein in PAQR3 overexpressed/interfered cells. **(C,D)** The effect of PAQR3 overexpression/inhibition on the proliferation of NSCLC cells were detected by CCK-8 assay. **(E,F)** The effect of PAQR3 overexpression/inhibition on the clone formation of NSCLC cells were assessed via clone formation assay. NSCLC, non-small cell lung cancer; Vector, overexpression control group; PAQR3-OV, PAQR3 overexpression group; Si-NC, knockdown control group; Si-PAQR3, PAQR3 knockdown group; *P < 0.05; **P < 0.01; ***P < 0.001; vs. relative control group.

**FIGURE 5:**
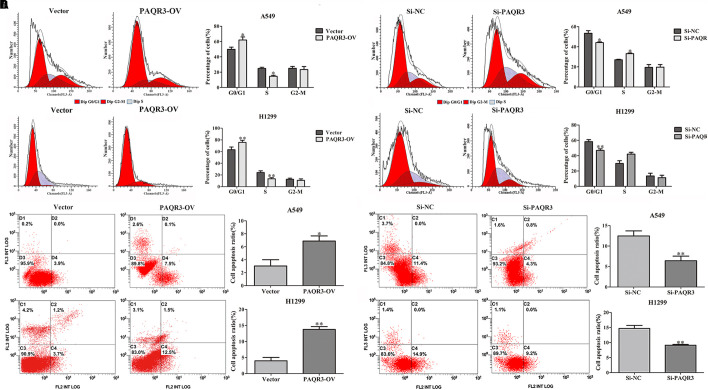
PAQR3 inhibited the cell cycle, but promoted apoptosis of NSCLC cells. **(A,B)** The effect of PAQR3 overexpression on cell cycle and apoptosis of NSCLC were detected by flow cytometry. **(C,D)** The effect of PAQR3 interference on cell cycle and apoptosis of NSCLC were detected by flow cytometry. NSCLC, non-small cell lung cancer; Vector, overexpression control group; PAQR3-OV, PAQR3 overexpression group; Si-NC, knockdown control group; Si-PAQR3, PAQR3 knockdown group; *P < 0.05; **P < 0.01; vs. relative control group.

Here, we used lentivirus to transfect NSCLC A549 and H1299 cells to construct PAQR3-overexpressed and PAQR3-interfered stable cells and found that the levels of PAQR3 mRNA and protein were significantly increased or decreased, respectively (Figures 4A,B). The results of the CCK-8 assay showed that increased PAQR3 expression significantly inhibited the proliferation and clone number, while decreased PAQR3 expression had the opposite effect (Figures 4C–F). In addition, flow cytometry analysis showed that overexpression of PAQR3 significantly induced the transition from the G1 to S phase and promoted apoptosis of NSCLC cells (Figures 5A,B), whereas interfering with PAQR3 yielded opposite outcomes (Figures 5C,D).

### GO and KEGG Enrichment Analyses Revealed Pathways Regulated by PAQR3 in NSCLC

**FIGURE 6:**
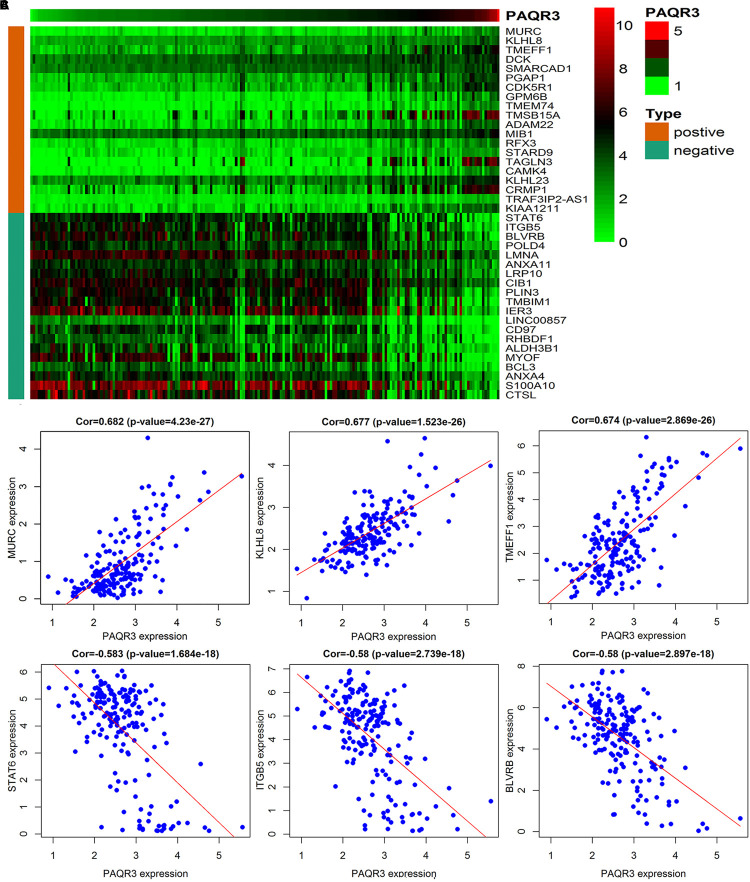
The top 20 positive and negative genes related to PAQR3. **(A)** The heat map of the top 20 positively and negatively related genes; **(B,C)** The top 3 positively and negatively related genes. positive, positively related genes; negative, negatively related genes; Cor, correlation coefficient.

**FIGURE 7:**
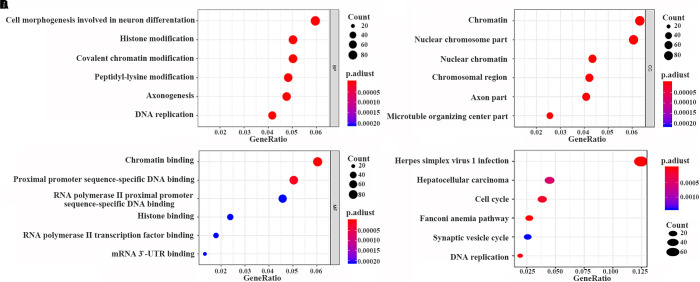
PAQR3 co-expressed genes analyzed by GO and KEGG with clusterProfiler software package on the R platform. **(A)** BP; **(B)** CC; **(C)** MF; **(D)** KEGG. BP, biological process; MF, molecular function; CC, cellular component; GO, gene ontology; KEGG, Kyoto Encyclopedia of Genes and Genomes.

**FIGURE 8:**
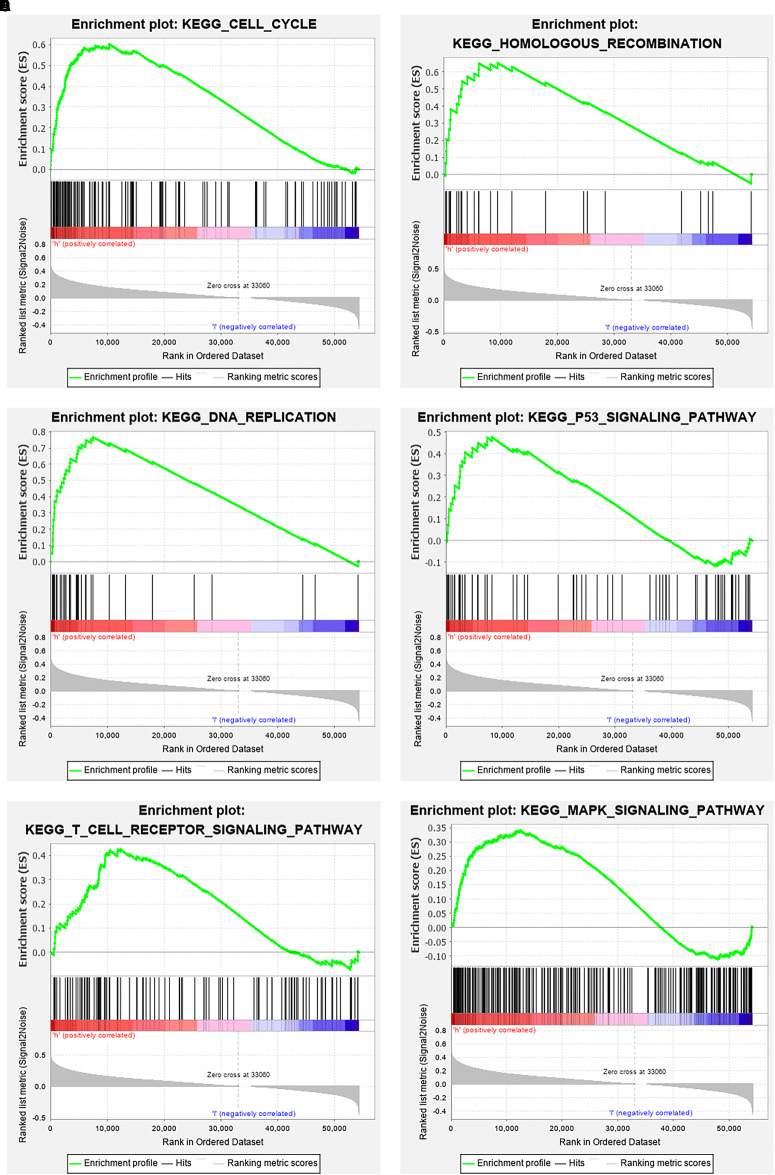
GSEA analysis revealed related signaling pathways enriched in PAQR3 high expression. **(A)** Cell cycle; **(B)** Homologous recombination; **(C)** DNA replication; **(D)** p53 signaling pathway; **(E)** T cell receptor signaling pathway; **(F)** MAPK signaling pathway. GSEA, gene set enrichment analysis.

The data were downloaded from the CCLE database, and 2015 co-expressed genes were screened (Supplementary Table S3). Among them, 1673 positively- and 342 negatively related genes were included. Figure 6 shows the top 20 positively- and negatively related PAQR3 co-expressed genes. GO annotations showed that PAQR3 co-expressed genes were mainly involved in DNA replication, DNA binding, RNA transport, etc (Figures 7A–C and Supplementary Table S4). In addition, KEGG analysis showed that they were mainly involved in the regulation of the cell cycle, DNA replication, Fanconi anemia pathway, and some other processes (Figure 7D). Second, it was revealed that increased PAQR3 expression was associated with cell cycle, DNA replication, homologous recombination, and the p53 signaling pathway through GSEA (NOM *p* < 0.05) (Figure 8 and Table 2). Therefore, this could suggest that PAQR3 might regulate the progress of NSCLC through the cell cycle, DNA replication, homologous recombination, and p53 signaling pathway.

**TABLE 2:** The possible physiological processes regulated by PAQR3 in lung cancer via GSEA.

Name	Size	ES	NES	NOM *p*
KEGG_CELL_CYCLE	124	0.603	2.062	0.002
KEGG_CHRONIC_MYELOID_LEUKEMIA	73	0.503	1.920	0.000
KEGG_PYRIMIDINE_METABOLISM	97	0.513	1.918	0.002
KEGG_DNA_REPLICATION	36	0.766	1.917	0.002
KEGG_UBIQUITIN_MEDIATED_PROTEOLYSIS	130	0.490	1.914	0.000
KEGG_SPLICEOSOME	124	0.624	1.886	0.015
KEGG_PROGESTERONE_MEDIATED_OOCYTE_MATURATION	85	0.517	1.882	0.000
KEGG_COLORECTAL_CANCER	62	0.501	1.872	0.000
KEGG_BASAL_TRANSCRIPTION_FACTORS	34	0.605	1.870	0.000
KEGG_BASE_EXCISION_REPAIR	34	0.596	1.867	0.004
KEGG_PHOSPHATIDYLINOSITOL_SIGNALING_SYSTEM	76	0.495	1.851	0.000
KEGG_HOMOLOGOUS_RECOMBINATION	26	0.651	1.823	0.012
KEGG_RNA_DEGRADATION	54	0.579	1.823	0.008
KEGG_MISMATCH_REPAIR	23	0.693	1.817	0.006
KEGG_NEUROTROPHIN_SIGNALING_PATHWAY	125	0.458	1.816	0.002
KEGG_P53_SIGNALING_PATHWAY	65	0.476	1.800	0.004
KEGG_ERBB_SIGNALING_PATHWAY	86	0.456	1.780	0.002
KEGG_RNA_POLYMERASE	29	0.619	1.779	0.020
KEGG_GAP_JUNCTION	88	0.444	1.758	0.000
KEGG_FC_EPSILON_RI_SIGNALING_PATHWAY	78	0.460	1.729	0.000
KEGG_OOCYTE_MEIOSIS	111	0.462	1.728	0.010
KEGG_GLIOMA	65	0.456	1.716	0.004
KEGG_NON_SMALL_CELL_LUNG_CANCER	54	0.464	1.706	0.008
KEGG_LONG_TERM_POTENTIATION	70	0.462	1.678	0.006
KEGG_T_CELL_RECEPTOR_SIGNALING_PATHWAY	107	0.425	1.676	0.008
KEGG_LYSINE_DEGRADATION	39	0.516	1.674	0.015
KEGG_PANCREATIC_CANCER	69	0.428	1.666	0.004
KEGG_TYPE_II_DIABETES_MELLITUS	47	0.480	1.665	0.006
KEGG_NUCLEOTIDE_EXCISION_REPAIR	44	0.535	1.664	0.027
KEGG_INSULIN_SIGNALING_PATHWAY	137	0.399	1.651	0.004
KEGG_MTOR_SIGNALING_PATHWAY	51	0.466	1.642	0.014
KEGG_PURINE_METABOLISM	153	0.412	1.620	0.028
KEGG_PROSTATE_CANCER	89	0.406	1.616	0.012
KEGG_ACUTE_MYELOID_LEUKEMIA	57	0.432	1.605	0.016
KEGG_LONG_TERM_DEPRESSION	67	0.433	1.602	0.006
KEGG_TASTE_TRANSDUCTION	51	0.593	1.592	0.008
KEGG_AXON_GUIDANCE	128	0.398	1.575	0.012
KEGG_VEGF_SIGNALING_PATHWAY	75	0.414	1.570	0.006
KEGG_RENAL_CELL_CARCINOMA	66	0.428	1.559	0.019
KEGG_FC_GAMMA_R_MEDIATED_PHAGOCYTOSIS	93	0.392	1.548	0.014
KEGG_INOSITOL_PHOSPHATE_METABOLISM	54	0.424	1.543	0.020
KEGG_GNRH_SIGNALING_PATHWAY	100	0.395	1.541	0.010
KEGG_ENDOMETRIAL_CANCER	52	0.430	1.530	0.027
KEGG_MAPK_SIGNALING_PATHWAY	265	0.341	1.514	0.004
KEGG_WNT_SIGNALING_PATHWAY	150	0.346	1.489	0.018
KEGG_EPITHELIAL_CELL_SIGNALING_IN_HELICOBACTER_PYLORI_INFECTION	67	0.387	1.478	0.019
KEGG_CHEMOKINE_SIGNALING_PATHWAY	184	0.340	1.430	0.023
KEGG_GLYCEROLIPID_METABOLISM	43	0.403	1.420	0.049

*ES, enrichment score; NES, normalized enrichment score; NOM p, nominal p-value.*

### PAQR3 Inhibited the Growth of NSCLC Through the NF-κB/p53/Bax Signaling Pathway

**FIGURE 9:**
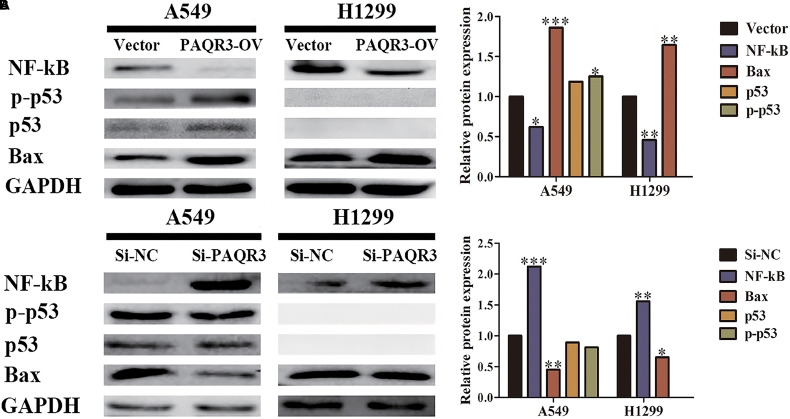
The effect of PAQR3 on NF-κB/p53/Bax signaling pathway was detected by western blotting. **(A)** Overexpression of PAQR3 inhibited the expression of NF-κB, but increased those of p53, phosphorylated p53, and Bax. **(B)** Knockdown of PAQR3 increased the expression of NF-κB protein, but inhibited those of p53, phosphorylated p53 and Bax. Vector, overexpression control group; PAQR3-OV, PAQR3 overexpression group; Si-NC, knockdown control group; Si-PAQR3, PAQR3 knockdown group; *P < 0.05; **P < 0.01; ***P < 0.001; vs. relative control group.

The effect of PAQR3 on the p53 signaling pathway was analyzed. In the stable transfection model of A549 and H1299 cells with overexpressed or silenced PAQR3, we found that overexpression of PAQR3 inhibited the expression of NF-κB protein and increased the expression of p53, p-p53, and Bax protein in A549 cells. Overexpression of PAQR3 inhibited the expression of NF-κB protein and increased the expression of Bax protein in H1299 cells, while no expression of p53 protein and p53 phosphorylation was detected (Figure 9A). However, interfering with the expression of PAQR3 in A549 cells promoted the expression of NF-κB protein and inhibited the expression of p53 protein, p53 phosphorylation, and Bax protein. Interfering with the expression of PAQR3 in H1299 cells enhanced the expression of NF-κB protein and inhibited the expression of Bax protein (Figure 9B).

## Discussion

A large number of studies have found that differentially expressed genes were potential biomarkers for prognosis in NSCLC patients. For instance, Han et al. found that the level of tripartite motif containing 47 (TRIM47) was increased in cancer tissues and closely related to poor prognosis in NSCLC. In addition, multivariate Cox regression analysis also showed that TRIM47 was an independent prognostic factor (Han et al., 2017). Liu et al. (2016) found that the levels of DEK in the cancer tissues were significantly upregulated and correlated with the pathological stage, disease-free survival, and OS time in NSCLC. The Cox proportional hazard regression model showed that DEK expression was an independent risk factor that affected prognosis (Liu et al., 2016). In addition, Schmidt et al. (2015) found that the level of PD-L1 in NSCLC was associated with tumor classification, tumor enlargement, positive lymph node, and prognosis of patients who received adjuvant therapy.

At present, the function of PAQR3 in cancers appears to be controversial. The level of PAQR3 was decreased both in tissues and cells of esophageal cancer, and overexpression of PAQR3 significantly inhibited the proliferation, clone formation, and invasion of esophageal cancer cells and blocked the transition from the G1 phase to the S phase. Moreover, increased PAQR3 expression inhibited the growth of ECA-109 cell xenograft tumors (Zhou et al., 2017; Bai et al., 2017, 2018). Compared with adjacent tissues, the expression of PAQR3 in laryngeal squamous cell carcinoma (LSCC) tissues was significantly downregulated, and overexpression of PAQR3 inhibited the proliferation and invasion of LSCC cells (Wu et al., 2016). In osteosarcoma, the expression of PAQR3 was decreased and was associated with metastasis in patients. In addition, overexpression of PAQR3 inhibited the proliferation, migration, and invasion of osteosarcoma MG-63 cells by promoting the phosphorylation of ERK (Ma et al., 2015). In the present study, we found that the level of PAQR3 mRNA in NSCLC was increased in cancer tissues analyzed using data from the Oncomine, Timer, and Ualcan databases, which were inconsistent with the conclusions reported by Liang et al. (2017) and Li et al. (2018). Therefore, we further determined the expression of PAQR3 in 60 clinical tissues by RT-qPCR and western blotting. We found that the levels of PAQR3 mRNA and protein were both decreased in most NSCLC patients. Furthermore, the level of PAQR3 mRNA was correlated with the histological subtype, lymph node metastasis, and tumor size, and was vital in the diagnosis of both NSCLC and its subtypes. In the databases related to prognosis, higher levels of PAQR3 predicted better prognosis for patients with LUAD. Therefore, we concluded that PAQR3 acted as a tumor suppressor in the progression of NSCLC and was expected to be a potential marker for prognosis and diagnosis in NSCLC.

p53 is an important tumor suppressor that plays vital roles in cell growth, cell cycle, apoptosis, angiogenesis, and genomic stability (Hiyama et al., 1998; Robles and Harris, 2001; Xu et al., 2014; Katifelis et al., 2018; Pfaff et al., 2018). In the process of tumorigenesis and development, increased p53 expression blocked the transformation of the cell cycle and promoted apoptosis. Through GSEA analysis, we found that increased PAQR3 expression was significantly correlated with the cell cycle, DNA replication, p53 signaling pathway, and some other processes of lung cancer. NF-κB and Bax proteins are the upstream and downstream targets of p53, respectively, and play significant roles in cell growth (Barone et al., 2012; Liang and Zhang, 2016; Sun et al., 2018; Chen W. et al., 2019). Moreover, Chen L. et al. (2019) found that PAQR3 regulated the phosphorylation of FoxO1 through the NF-κB signaling pathway in insulin-resistant HepG2 cells. In the present study, we found that increased PAQR3 expression in A549 cells inhibited NF-κB (P65) protein expression and increased p53 protein, p53 phosphorylation, and Bax protein expression. Increased PAQR3 expression in H1299 cells inhibited NF-κB expression and promoted Bax protein expression, while p53 protein and p53 phosphorylation expression were not affected. Opposite results would be present when PAQR3 was knocked down. Both A549 and H1299 cells are LUAD cells, the difference being that p53 protein is absent in H1299 cells. Through GSEA and model construction, we found that NF-κB, Bax, and p53 were changed in A549 cells, while the expression levels of p53 protein and p53 phosphorylation protein in H1299 cells had no effect. Only the NF-κB and Bax proteins changed, which were expected results. These results suggest that PAQR3 acts as a tumor suppressor in the occurrence and development of NSCLC through the NF-kB/p53/Bax signaling pathway.

Compared with previous studies, our research is more comprehensive. Using various databases, we found that the expression of PAQR3 was increased in LUSC and LUAD tissues, and verified the expression of PAQR3 in clinical NSCLC tissues by RT-qPCR and western blotting. At the cellular level, a new clone formation assay was added to verify the effect of PAQR3 on the growth of NSCLC cells. In addition, we used GSEA to evaluate whether PAQR3 may regulate the progression of lung cancer through the p53 signaling pathway and confirmed our prediction in model cells. However, our study still had several shortcomings, which warrant that we further supplement the relevant results of PAQR3 in nude mice. In summary, the expressions of PAQR3 mRNA and protein were downregulated and was significantly related to tumor size, histological subtype, and lymph node metastasis in NSCLC patients. Moreover, it could be a positive biomarker for prognosis among patients with LUAD. In addition, PAQR3 inhibited the growth through the NF-κB/p53/Bax signaling pathway to participate in regulating the progression of NSCLC.

## Data Availability Statement

All datasets presented in this study are included in the article/Supplementary Material.

## Ethics Statement

The studies involving human participants were reviewed and approved by Ethics Committee of the Affiliated Hospital of Zunyi Medical University (KLLY-2018-095). The patients/participants provided their written informed consent to participate in this study.

## Author Contributions

GX, H-LL, and CC designed the study. QG and X-XK performed the experiments. S-XF, W-LG, Y-XS, and CC supervised the project. CC and X-XK analyzed the data. QG drafted the manuscript, which was corrected by GX and H-LL. All authors contributed to completing this study, read, and approved the final manuscript.

## Conflict of Interest

The authors declare that the research was conducted in the absence of any commercial or financial relationships that could be construed as a potential conflict of interest.

## Footnotes

1 https://www.oncomine.org/resource/login

2 https://cistrome.shinyapps.io/timer/

3 http://ualcan.path.uab.edu

4 http://www.abren.net/PrognoScan/

5 http://kmplot.com/analysis/
