# Supplementary material for: PAQR3 Inhibits Non-small Cell Lung Cancer Growth by Regulating the NF-κB/p53/Bax Axis
Source: Front Cell Dev Biol. 2020 Oct 6;8:581919. doi: 10.3389/fcell.2020.581919 (PMC7573313; doi:10.3389/fcell.2020.581919)
Supplement: Supplementary file 1 [file Data_Sheet_1.docx]

Supplementary Material

**
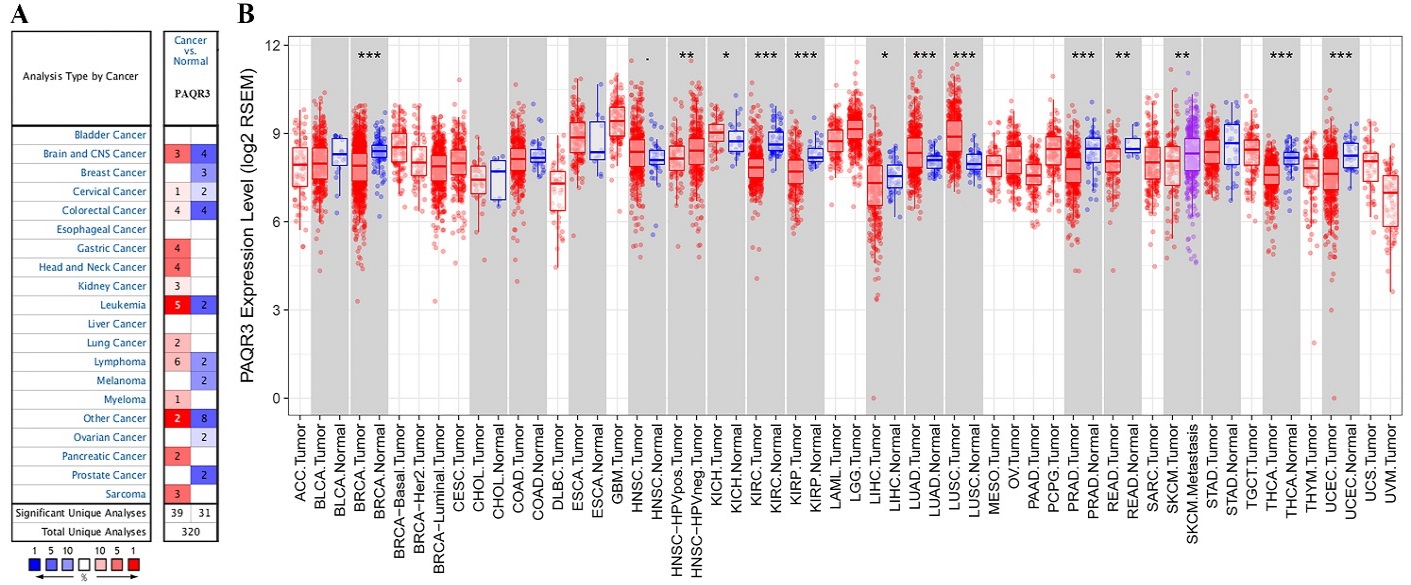
**

**Figure S1.** PAQR3 mRNA expression in NSCLC from Oncomine and Timer databases. (A) Oncomine; (B) Timer. Note: (A) Red indicates increased expression of PAQR3, and blue indicates decreased expression; (B) Red indicates tumor tissue, blue indicates normal tissue; *, *P* < 0.05; **, *P* < 0.01; ***, *P* < 0.001 vs normal tissue.


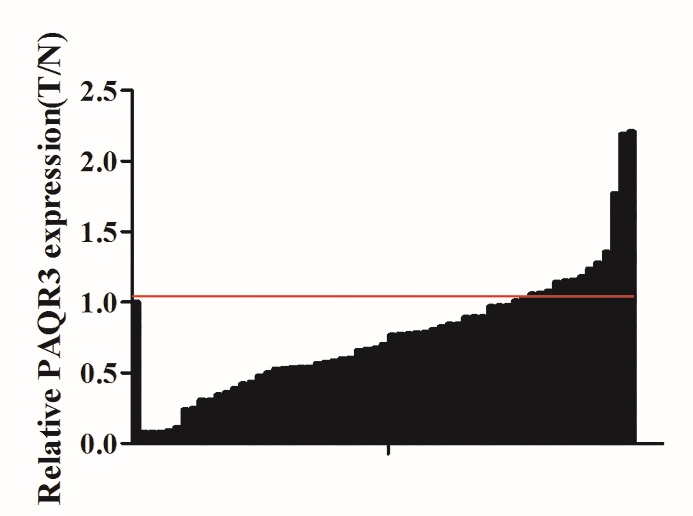


**Figure S2.** Relative expression of PAQR3 protein in NSCLC tissue compared with adjacent normal lung tissue. Note: N, normal lung tissue; T, non-small cell lung cancer tissue.


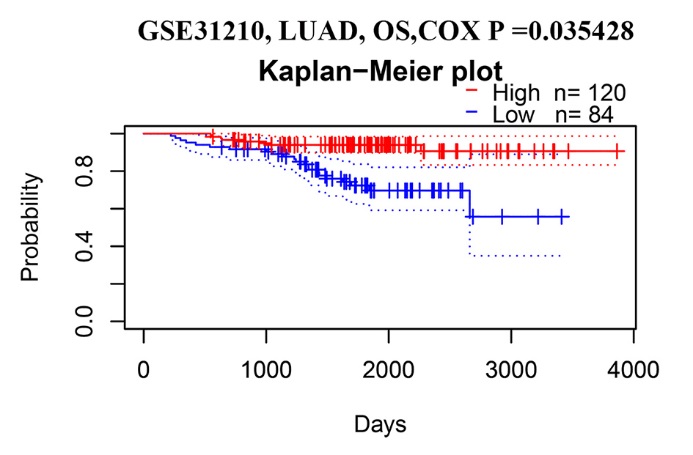


**Figure S3.** In the PrognoScan database, LUAD patients with increased PAQR3 tended to have better prognoses.

Table S1. General data of patients with non-small cell lung cancer in Ualcan database

| Clinicopathologic features | N | Percentage (%) |
| --- | --- | --- |
| Age |  |  |
| ≦60 | 207 | 26.9 |
| >60 | 562 | 73.1 |
| Gender |  |  |
| Male | 604 | 59.9 |
| Female | 404 | 40.1 |
| Race |  |  |
| Caucasian | 730 | 88.2 |
| African-american | 81 | 9.8 |
| Asian | 17 | 2.0 |
| Smoking |  |  |
| No | 93 | 9.5 |
| Yes | 884 | 90.5 |
| Histological type |  |  |
| LUSC | 503 | 49.4 |
| LUAD | 515 | 50.6 |
| Clinical stage |  |  |
| I-II | 802 | 79.6 |
| III-IV | 205 | 20.4 |
| Lymph node metastasis |  |  |
| No | 651 | 65.2 |
| Yes | 348 | 34.8 |

Note: LUAD, lung adenocarcinoma; LUSC, lung squamous carcinoma.

Table S2. General data of 60 patients with non-small cell lung cancer

| Clinicopathologic | N | Percentage (%) |
| --- | --- | --- |
| Age |  |  |
| ≦60 | 27 | 45 |
| >60 | 33 | 55 |
| Gender |  |  |
| Male | 28 | 46.7 |
| Female | 32 | 53.3 |
| Smoking |  |  |
| No | 40 | 66.7 |
| Yes | 20 | 33.3 |
| Histological type |  |  |
| LUSC | 30 | 50 |
| LUAD | 30 | 50 |
| Tumor size |  |  |
| ≦5 | 28 | 46.7 |
| >5 | 32 | 53.3 |
| Clincial stage |  |  |
| I-II | 47 | 78.3 |
| III | 13 | 11.7 |
| T stage |  |  |
| T1-T2 | 45 | 75 |
| T3-T4 | 15 | 25 |
| Lymph node metastasis |  |  |
| No | 43 | 71.7 |
| Yes | 17 | 28.3 |

Note: LUAD, lung adenocarcinoma; LUSC, lung squamous carcinoma.

Table S3. Positive and negative genes related to PAQR3 in lung cancer cells

| Gene | Cor | Gene | Cor | Gene | Cor |  | Gene | Cor |
| --- | --- | --- | --- | --- | --- | --- | --- | --- |
| MURC | 0.682 | ZMYM3 | 0.489 | DPY19L2 | 0.442 |  | GPR19 | 0.41 |
| KLHL8 | 0.677 | RP13-36G14.4 | 0.489 | TMPO | 0.442 |  | PDS5B | 0.41 |
| TMEFF1 | 0.674 | CEP68 | 0.488 | RPS6KL1 | 0.442 |  | RP11-521B24.3 | 0.41 |
| DCK | 0.664 | XPO7 | 0.488 | STX1B | 0.442 |  | RP11-797A18.6 | 0.41 |
| SMARCAD1 | 0.641 | RUNDC3A | 0.488 | ZNF821 | 0.442 |  | AC005943.5 | 0.41 |
| PGAP1 | 0.64 | SAMD14 | 0.488 | ZNF491 | 0.442 |  | MBD3 | 0.41 |
| CDK5R1 | 0.639 | ZNF625 | 0.488 | RNASEH2A | 0.442 |  | ASPDH | 0.41 |
| GPM6B | 0.636 | HUNK | 0.488 | ZNF428 | 0.442 |  | NINL | 0.41 |
| TMEM74 | 0.625 | EPB41 | 0.487 | GAS5-AS1 | 0.441 |  | FOXJ3 | 0.409 |
| TMSB15A | 0.617 | FAM212B | 0.487 | PAPOLG | 0.441 |  | NHLH2 | 0.409 |
| ADAM22 | 0.613 | ZNF142 | 0.487 | RP11-362K14.6 | 0.441 |  | E2F3 | 0.409 |
| MIB1 | 0.613 | RP11-398K22.12 | 0.487 | RCHY1 | 0.441 |  | CAMK2B | 0.409 |
| RFX3 | 0.611 | MANEA | 0.487 | C11orf30 | 0.441 |  | KAT6A | 0.409 |
| STARD9 | 0.611 | HDAC2 | 0.487 | ZNF664 | 0.441 |  | TOPORS | 0.409 |
| TAGLN3 | 0.61 | LONRF1 | 0.487 | PROSER1 | 0.441 |  | DCAF12 | 0.409 |
| CAMK4 | 0.609 | FBXO10 | 0.487 | CHGA | 0.441 |  | SLC25A51 | 0.409 |
| KLHL23 | 0.607 | NELL2 | 0.487 | RP11-649A18.4 | 0.441 |  | VWCE | 0.409 |
| CRMP1 | 0.607 | RLTPR | 0.487 | C17orf70 | 0.441 |  | C12orf76 | 0.409 |
| TRAF3IP2-AS1 | 0.607 | RAB3A | 0.487 | ZNF407 | 0.441 |  | IFT81 | 0.409 |
| KIAA1211 | 0.606 | SVOP | 0.486 | PASK | 0.44 |  | KBTBD6 | 0.409 |
| HNRNPD | 0.603 | C16orf59 | 0.486 | POLR2B | 0.44 |  | TFDP1 | 0.409 |
| CTD-2006C1.6 | 0.603 | SAMD1 | 0.486 | SKP2 | 0.44 |  | LINC00641 | 0.409 |
| CCP110 | 0.6 | ZBTB33 | 0.486 | STARD4-AS1 | 0.44 |  | ZNF527 | 0.409 |
| FSD1L | 0.596 | RIMS3 | 0.485 | USP45 | 0.44 |  | MFSD2A | 0.408 |
| ELAVL3 | 0.596 | PRPF38A | 0.485 | MICAL1 | 0.44 |  | MAST2 | 0.408 |
| PSIP1 | 0.595 | DTL | 0.485 | RP11-1C8.7 | 0.44 |  | CYB5RL | 0.408 |
| GPR63 | 0.594 | GNG4 | 0.485 | TENM4 | 0.44 |  | LRRC40 | 0.408 |
| RTN1 | 0.593 | TSPYL4 | 0.485 | DRD2 | 0.44 |  | PELI1 | 0.408 |
| KLF12 | 0.591 | EP400 | 0.485 | ATL1 | 0.44 |  | GNMT | 0.408 |
| C9orf72 | 0.59 | NOVA1 | 0.485 | RP11-57A19.2 | 0.44 |  | ZNRF2P2 | 0.408 |
| MAP6 | 0.59 | ZNF24 | 0.485 | RP5-837J1.2 | 0.44 |  | GATS | 0.408 |
| KIF5C | 0.588 | SNAP25 | 0.485 | RNF165 | 0.44 |  | CER1 | 0.408 |
| RNF138 | 0.588 | CLCN5 | 0.485 | RP4-657E11.10 | 0.439 |  | RP11-195F19.5 | 0.408 |
| TCF4 | 0.588 | ARMCX4 | 0.485 | GUSBP9 | 0.439 |  | KIAA1731 | 0.408 |
| HRH3 | 0.588 | RPL18AP7 | 0.484 | REEP2 | 0.439 |  | TIGD7 | 0.408 |
| ZNF445 | 0.586 | YEATS2 | 0.484 | NFYA | 0.439 |  | HDAC5 | 0.408 |
| RAMP2-AS1 | 0.585 | UBXN7 | 0.484 | KIAA1586 | 0.439 |  | ANKRD40 | 0.408 |
| KIAA0408 | 0.584 | SRRM3 | 0.484 | B3GAT2 | 0.439 |  | MKS1 | 0.408 |
| STMN1 | 0.583 | NUP160 | 0.484 | RP11-531A24.3 | 0.439 |  | SEH1L | 0.408 |
| SOWAHA | 0.583 | SEPT4 | 0.484 | ZDHHC17 | 0.439 |  | CHST9 | 0.408 |
| IGFBPL1 | 0.583 | MIAT | 0.484 | SYNRG | 0.439 |  | ZNF304 | 0.408 |
| USP49 | 0.582 | NASP | 0.483 | KPNB1 | 0.439 |  | TRIM33 | 0.407 |
| HNRNPDL | 0.581 | RTN3 | 0.483 | AC004447.2 | 0.439 |  | NAV1 | 0.407 |
| THAP9 | 0.581 | MAPT | 0.483 | SUV420H2 | 0.439 |  | UBE2T | 0.407 |
| C18orf54 | 0.581 | CBX1 | 0.483 | PCSK2 | 0.439 |  | GREB1 | 0.407 |
| CECR6 | 0.579 | PIK3C3 | 0.483 | WRAP73 | 0.438 |  | PCNP | 0.407 |
| RP1-257A7.5 | 0.578 | ZNF551 | 0.483 | FOXO6 | 0.438 |  | PCMT1 | 0.407 |
| SOGA3 | 0.578 | GDAP1L1 | 0.483 | MLLT11 | 0.438 |  | ELMO1 | 0.407 |
| MEX3B | 0.578 | MCM3AP-AS1 | 0.483 | NFASC | 0.438 |  | FEZF1 | 0.407 |
| GPR173 | 0.578 | C1orf21 | 0.482 | WDR48 | 0.438 |  | PTENP1 | 0.407 |
| ZCCHC18 | 0.578 | NAB1 | 0.482 | ZNF141 | 0.438 |  | KCNIP2 | 0.407 |
| CREB1 | 0.577 | BEND3 | 0.482 | RNF8 | 0.438 |  | BEST3 | 0.407 |
| PCYT1B | 0.577 | ZBTB5 | 0.482 | FRS3 | 0.438 |  | EVL | 0.407 |
| UNC79 | 0.576 | RP11-213G2.3 | 0.482 | CCT6P1 | 0.438 |  | SLC25A29 | 0.407 |
| MAST1 | 0.575 | CHRM4 | 0.482 | ZNF84 | 0.438 |  | INTS2 | 0.407 |
| URB2 | 0.574 | CKAP5 | 0.482 | ZMYM2 | 0.438 |  | CDH2 | 0.407 |
| ACVR2B | 0.574 | CEP152 | 0.482 | RAVER1 | 0.438 |  | KIAA1468 | 0.407 |
| CAMTA1 | 0.573 | PATZ1 | 0.482 | CACNA1A | 0.438 |  | EID2B | 0.407 |
| GS1-279B7.2 | 0.573 | ZNF670 | 0.481 | ZNF724P | 0.438 |  | PTOV1-AS1 | 0.407 |
| DPYSL5 | 0.573 | NRXN1 | 0.481 | FAM168B | 0.437 |  | RP4-769N13.6 | 0.407 |
| ALMS1 | 0.573 | WDFY3 | 0.481 | KIAA0226 | 0.437 |  | RP1-283E3.4 | 0.406 |
| PHACTR1 | 0.573 | MATR3 | 0.481 | ZNF184 | 0.437 |  | H1FX | 0.406 |
| SH3GL2 | 0.573 | MMS22L | 0.481 | GCK | 0.437 |  | ANK1 | 0.406 |
| TTBK1 | 0.572 | ARHGEF7 | 0.481 | ATP6V1B2 | 0.437 |  | ZBTB34 | 0.406 |
| BRSK1 | 0.572 | TOP3A | 0.481 | UBXN2B | 0.437 |  | C2CD2L | 0.406 |
| ATP1B3-AS1 | 0.571 | ST8SIA5 | 0.481 | PHRF1 | 0.437 |  | INHBE | 0.406 |
| TMOD2 | 0.571 | AP001469.9 | 0.481 | ARGLU1 | 0.437 |  | AC002310.12 | 0.406 |
| ACTL6B | 0.57 | KLHL32 | 0.48 | RP11-540B6.6 | 0.437 |  | MED13 | 0.406 |
| BEND5 | 0.569 | DGKI | 0.48 | MAP1A | 0.437 |  | SUMO2 | 0.406 |
| KBTBD8 | 0.569 | ZNF136 | 0.48 | MFAP4 | 0.437 |  | PRR14L | 0.406 |
| C7orf41 | 0.569 | VBP1 | 0.48 | ZNF611 | 0.437 |  | USP9X | 0.406 |
| EBF3 | 0.569 | TMEM57 | 0.479 | UBE2S | 0.437 |  | LL0XNC01-7P3.1 | 0.406 |
| RNF219 | 0.568 | RP11-91J19.4 | 0.479 | PHF6 | 0.437 |  | GPRASP2 | 0.406 |
| HMGN2P15 | 0.567 | STMN2 | 0.479 | FAM72A | 0.436 |  | RP1-315G1.3 | 0.406 |
| CENPC | 0.565 | CNTLN | 0.479 | SOS1 | 0.436 |  | RP11-169K16.9 | 0.405 |
| DCHS1 | 0.565 | KIAA1467 | 0.479 | DLX1 | 0.436 |  | RP11-317P15.5 | 0.405 |
| NCAM1 | 0.565 | KDM2B | 0.479 | WDFY3-AS2 | 0.436 |  | HOXD-AS1 | 0.405 |
| TMTC4 | 0.565 | RP11-74E22.4 | 0.479 | DLX6 | 0.436 |  | NR2C2 | 0.405 |
| KIDINS220 | 0.564 | UNC13A | 0.479 | LINC00938 | 0.436 |  | DBR1 | 0.405 |
| SYN1 | 0.564 | TUBA3FP | 0.479 | BPTF | 0.436 |  | FAM193A | 0.405 |
| AC011306.2 | 0.563 | KLHL15 | 0.479 | NARF | 0.436 |  | RFC1 | 0.405 |
| TMEM108 | 0.563 | RP4-635E18.7 | 0.478 | AP000251.3 | 0.436 |  | ANKRD18EP | 0.405 |
| JAKMIP2 | 0.563 | ZYG11B | 0.478 | CROCCP3 | 0.435 |  | RP1-8B1.4 | 0.405 |
| CEP97 | 0.562 | DNAJC18 | 0.478 | EFCAB7 | 0.435 |  | RBM4B | 0.405 |
| SHPRH | 0.562 | RUNX1T1 | 0.478 | FBXO41 | 0.435 |  | RFC5 | 0.405 |
| HAUS6 | 0.562 | ZDHHC21 | 0.478 | RP11-477D19.2 | 0.435 |  | COCH | 0.405 |
| PHF21B | 0.562 | C11orf84 | 0.478 | LINC00599 | 0.435 |  | RP11-22P6.3 | 0.405 |
| TRO | 0.562 | ZNF519 | 0.478 | AMER2 | 0.435 |  | SSTR2 | 0.405 |
| OTUD3 | 0.561 | ADNP2 | 0.478 | FAM155A | 0.435 |  | RNMT | 0.405 |
| HMGN2 | 0.561 | TTC3 | 0.478 | DLK1 | 0.435 |  | RPL23AP79 | 0.405 |
| AC092835.2 | 0.561 | TRAPPC2 | 0.478 | RP11-680G10.1 | 0.435 |  | ZCCHC12 | 0.405 |
| ZNF620 | 0.561 | RP4-798A10.2 | 0.477 | KCNAB3 | 0.435 |  | HNRNPU | 0.404 |
| SP4 | 0.561 | TADA1 | 0.477 | RHBDL3 | 0.435 |  | R3HDM1 | 0.404 |
| MSI1 | 0.561 | EIF3EP1 | 0.477 | LUC7L3 | 0.435 |  | ARMC8 | 0.404 |
| RIF1 | 0.56 | DPY19L2P2 | 0.477 | HDGFRP2 | 0.435 |  | NMNAT3 | 0.404 |
| RAB39B | 0.56 | GKAP1 | 0.477 | CTC-359D24.3 | 0.435 |  | RGS12 | 0.404 |
| IRAK1BP1 | 0.559 | MRPS31P5 | 0.477 | SULT4A1 | 0.435 |  | ZKSCAN8 | 0.404 |
| FXYD6 | 0.559 | DDHD1 | 0.477 | ANK2 | 0.434 |  | MICU3 | 0.404 |
| POLA1 | 0.559 | ZNF48 | 0.477 | ZNF853 | 0.434 |  | HECTD2 | 0.404 |
| HNRNPR | 0.558 | HELZ | 0.477 | RP11-115C21.2 | 0.434 |  | INCENP | 0.404 |
| EYA3 | 0.558 | ELP2 | 0.477 | POMK | 0.434 |  | ASCL1 | 0.404 |
| POU2F1 | 0.558 | DYM | 0.477 | NFIB | 0.434 |  | IPO5 | 0.404 |
| CBLN1 | 0.557 | PHF8 | 0.477 | CCNJ | 0.434 |  | CTCF | 0.404 |
| ST8SIA3 | 0.557 | ACAP2-IT1 | 0.476 | ARID2 | 0.434 |  | CTD-3193K9.3 | 0.404 |
| RP5-935K16.1 | 0.556 | RASL11B | 0.476 | MYCBP2 | 0.434 |  | DIRAS1 | 0.404 |
| GNAZ | 0.556 | MAP1B | 0.476 | SUPT16H | 0.434 |  | ZNF101 | 0.404 |
| RUFY3 | 0.555 | JPH4 | 0.476 | CORO2B | 0.434 |  | ZNF43 | 0.404 |
| FBXO5 | 0.555 | ZNF232 | 0.476 | MEF2A | 0.434 |  | GRIK5 | 0.404 |
| ELAVL4 | 0.554 | RP11-379L18.1 | 0.476 | ELK1 | 0.434 |  | ZNF548 | 0.404 |
| ITPRIPL1 | 0.554 | SGTA | 0.476 | COL9A2 | 0.433 |  | C21orf58 | 0.404 |
| RQCD1 | 0.554 | RP11-706O15.1 | 0.476 | HOXD3 | 0.433 |  | NPM1P9 | 0.404 |
| RASGEF1B | 0.554 | ATRX | 0.476 | XRN1 | 0.433 |  | FAM155B | 0.404 |
| SRSF12 | 0.554 | FAM76A | 0.475 | WDR19 | 0.433 |  | ZDHHC15 | 0.404 |
| FOXN4 | 0.554 | LRRC58 | 0.475 | XXbac-BPG252P9.9 | 0.433 |  | FBXO48 | 0.403 |
| INSM1 | 0.554 | ANKRD17 | 0.475 | MCPH1 | 0.433 |  | RABL2A | 0.403 |
| SMC1A | 0.554 | AP3B2 | 0.475 | RP11-3J10.4 | 0.433 |  | TMEFF2 | 0.403 |
| RAB9B | 0.554 | CTD-3193O13.9 | 0.475 | ATRNL1 | 0.433 |  | EAF1 | 0.403 |
| KIF5A | 0.553 | CTXN1 | 0.475 | NAA40 | 0.433 |  | SLC35D3 | 0.403 |
| MTMR4 | 0.553 | TP53BP2 | 0.474 | TMEM194A | 0.433 |  | PCLO | 0.403 |
| DPF1 | 0.553 | FANCL | 0.474 | FOXK2 | 0.433 |  | KCNH2 | 0.403 |
| PAXBP1 | 0.553 | ELF2 | 0.474 | SMCHD1 | 0.433 |  | GXYLT1 | 0.403 |
| FAM57B | 0.552 | FAM159B | 0.474 | RP11-490K7.4 | 0.432 |  | AKAP11 | 0.403 |
| LIMD2 | 0.552 | UBN2 | 0.474 | RP11-332H14.2 | 0.432 |  | ARG2 | 0.403 |
| TMSB15B | 0.552 | LINC00617 | 0.474 | USP13 | 0.432 |  | BCL11B | 0.403 |
| RBBP5 | 0.55 | CCSAP | 0.473 | USP46 | 0.432 |  | NLRP1 | 0.403 |
| PBRM1 | 0.55 | NUP133 | 0.473 | RAB3C | 0.432 |  | GDPD1 | 0.403 |
| TBPL1 | 0.55 | ZNF669 | 0.473 | ELOVL4 | 0.432 |  | ZNF516 | 0.403 |
| PSMC3IP | 0.55 | NREP | 0.473 | DYNC1I1 | 0.432 |  | TXNL4A | 0.403 |
| MEX3A | 0.549 | KSR2 | 0.473 | UBA52P6 | 0.432 |  | C22orf39 | 0.403 |
| G3BP2 | 0.549 | ZNF891 | 0.473 | SH2D3C | 0.432 |  | MTF2 | 0.402 |
| TMEM151B | 0.549 | ZNF562 | 0.473 | UCK1 | 0.432 |  | CENPF | 0.402 |
| CELF4 | 0.549 | ARHGAP33 | 0.473 | TUB | 0.432 |  | UBA6 | 0.402 |
| PKNOX1 | 0.549 | TAF9B | 0.473 | NELL1 | 0.432 |  | UBE2QL1 | 0.402 |
| USP37 | 0.548 | FBXO42 | 0.472 | NXF1 | 0.432 |  | FAM120B | 0.402 |
| RP3-337H4.8 | 0.548 | FMN2 | 0.472 | CAND1 | 0.432 |  | TRIM60P18 | 0.402 |
| KCNC1 | 0.548 | RIPPLY2 | 0.472 | ATP6V0A2 | 0.432 |  | RP11-87H9.2 | 0.402 |
| ENO2 | 0.548 | TMEM181 | 0.472 | GPR12 | 0.432 |  | CENPP | 0.402 |
| SNX22 | 0.548 | LIN7A | 0.472 | RAP2A | 0.432 |  | TSTD2 | 0.402 |
| KCNH4 | 0.548 | CCNF | 0.472 | WDR76 | 0.432 |  | PRRC2B | 0.402 |
| ELAVL1 | 0.548 | SBK1 | 0.472 | ASXL3 | 0.432 |  | RBM14 | 0.402 |
| TMCC2 | 0.546 | CXorf24 | 0.472 | ZADH2 | 0.432 |  | KCNH3 | 0.402 |
| RP11-381E24.1 | 0.546 | NKAIN1 | 0.471 | ZNF333 | 0.432 |  | FBRSL1 | 0.402 |
| FAM184A | 0.545 | CELF3 | 0.471 | ZNF738 | 0.432 |  | RBM26 | 0.402 |
| SALL2 | 0.545 | KCNH7 | 0.471 | TCEB3 | 0.431 |  | PRPF39 | 0.402 |
| PIAS2 | 0.545 | SYN2 | 0.471 | MAN1C1 | 0.431 |  | RP11-649A18.7 | 0.402 |
| PPAT | 0.544 | RP11-2E11.9 | 0.471 | MYT1L | 0.431 |  | C2CD4C | 0.402 |
| CCNI | 0.544 | RPL23AP53 | 0.471 | CEP135 | 0.431 |  | LSM14B | 0.402 |
| HIATL2 | 0.544 | RNPS1 | 0.471 | AEBP1 | 0.431 |  | AC006946.16 | 0.402 |
| HERC2P10 | 0.544 | DHPS | 0.471 | PCM1 | 0.431 |  | GPN2 | 0.401 |
| CLASP1 | 0.543 | AKT3 | 0.47 | RP11-598P20.3 | 0.431 |  | ZFP69 | 0.401 |
| NSG1 | 0.543 | MSH2 | 0.47 | JPH1 | 0.431 |  | RBM6 | 0.401 |
| POLE | 0.543 | RP11-348P10.2 | 0.47 | SSRP1 | 0.431 |  | CEP44 | 0.401 |
| GRM2 | 0.542 | MCF2L2 | 0.47 | PRDM10 | 0.431 |  | AMBRA1 | 0.401 |
| LCORL | 0.542 | LPHN3 | 0.47 | RP11-359B12.2 | 0.431 |  | SLC36A4 | 0.401 |
| HSF2 | 0.542 | RNF150 | 0.47 | SART3 | 0.431 |  | MED17 | 0.401 |
| RPRD1A | 0.542 | REV3L | 0.47 | CCNA1 | 0.431 |  | C2CD5 | 0.401 |
| SOX11 | 0.541 | NAP1L4 | 0.47 | TPP2 | 0.431 |  | TUBA1B | 0.401 |
| ARL6 | 0.541 | E2F7 | 0.47 | FOXG1 | 0.431 |  | H3F3BP1 | 0.401 |
| HACE1 | 0.541 | ZIC2 | 0.47 | VAT1L | 0.431 |  | AC005519.4 | 0.401 |
| PIANP | 0.541 | ZSCAN22 | 0.47 | DHX40 | 0.431 |  | NPIPP1 | 0.401 |
| PPM1E | 0.541 | RBM10 | 0.47 | CXXC1 | 0.431 |  | LIG3 | 0.401 |
| TXLNG | 0.541 | DNAJC6 | 0.469 | ZNF420 | 0.431 |  | MEX3C | 0.401 |
| RP11-33B1.1 | 0.54 | SPAST | 0.469 | GNG8 | 0.431 |  | C19orf82 | 0.401 |
| ATP6V1G2 | 0.54 | KANSL1L | 0.469 | ZNF776 | 0.431 |  | RBBP7 | 0.401 |
| SLCO5A1 | 0.54 | RP11-884K10.7 | 0.469 | DYRK1A | 0.431 |  | FOSL2 | -0.401 |
| RC3H2 | 0.54 | C4orf21 | 0.469 | SPEN | 0.43 |  | RIPK2 | -0.401 |
| DDX25 | 0.54 | GPR98 | 0.469 | KCNT2 | 0.43 |  | PROSER2 | -0.401 |
| LRRC16B | 0.54 | SCAF8 | 0.469 | NSL1 | 0.43 |  | TRIM5 | -0.401 |
| YPEL1 | 0.54 | LINGO2 | 0.469 | ESRRG | 0.43 |  | CAV1 | -0.402 |
| PHF16 | 0.54 | ACBD7 | 0.469 | SATB1 | 0.43 |  | MYO1C | -0.402 |
| SLC25A53 | 0.54 | CDCA5 | 0.469 | PCGF3 | 0.43 |  | SERTAD3 | -0.402 |
| SPSB4 | 0.539 | LINC00403 | 0.469 | C1QTNF3 | 0.43 |  | PLEKHN1 | -0.403 |
| CACNA1B | 0.539 | RND2 | 0.469 | CHN2 | 0.43 |  | MTMR11 | -0.403 |
| SMAD9 | 0.539 | AC007292.6 | 0.469 | IMMP1L | 0.43 |  | PQLC3 | -0.403 |
| DCUN1D2 | 0.539 | ARL6IP6 | 0.468 | PHF21A | 0.43 |  | SP110 | -0.403 |
| SEZ6 | 0.539 | ILKAP | 0.468 | NAALADL1 | 0.43 |  | GPX8 | -0.403 |
| ZNF711 | 0.539 | WHSC1 | 0.468 | RSF1 | 0.43 |  | IGFBP3 | -0.403 |
| RNF144A | 0.538 | SCRT1 | 0.468 | RCBTB1 | 0.43 |  | TAOK3 | -0.403 |
| FAM117B | 0.538 | N4BP2L1 | 0.468 | EXOC5 | 0.43 |  | RP11-46D6.1 | -0.403 |
| ENOPH1 | 0.538 | ZDHHC22 | 0.468 | ACYP1 | 0.43 |  | GRN | -0.403 |
| SNAP91 | 0.538 | CLIP3 | 0.468 | CELF6 | 0.43 |  | C20orf24 | -0.403 |
| NUPL1 | 0.538 | CNKSR2 | 0.468 | AC000068.5 | 0.43 |  | SLC35F6 | -0.404 |
| SEPT3 | 0.538 | HP1BP3 | 0.467 | KDM5C | 0.43 |  | RNF149 | -0.404 |
| CNIH2 | 0.537 | MEAF6 | 0.467 | RP4-635E18.8 | 0.429 |  | AC005355.2 | -0.404 |
| RAB39A | 0.537 | VASH2 | 0.467 | DYRK3 | 0.429 |  | PON2 | -0.404 |
| TUBA1A | 0.537 | WDR33 | 0.467 | KANSL3 | 0.429 |  | PSMB10 | -0.404 |
| PPM1D | 0.537 | WDR17 | 0.467 | ACVR2A | 0.429 |  | SLC10A3 | -0.404 |
| CTD-2619J13.16 | 0.537 | AC004540.4 | 0.467 | TMEM198 | 0.429 |  | EFNA4 | -0.405 |
| Z83851.1 | 0.537 | ZKSCAN2 | 0.467 | SETMAR | 0.429 |  | TTC7A | -0.405 |
| CDC7 | 0.536 | CBFA2T2 | 0.467 | CAMK2N2 | 0.429 |  | TBC1D2 | -0.405 |
| PARP1 | 0.536 | ASPHD2 | 0.467 | PRKG2 | 0.429 |  | GALE | -0.406 |
| CEP170 | 0.536 | ZNF326 | 0.466 | DCLK2 | 0.429 |  | GPR126 | -0.406 |
| ZNF324 | 0.536 | TMEM237 | 0.466 | PHF10 | 0.429 |  | ANXA7 | -0.406 |
| CITED1 | 0.536 | KALRN | 0.466 | DMTF1 | 0.429 |  | RILP | -0.406 |
| NACAD | 0.535 | DOPEY1 | 0.466 | ATP6V0E2-AS1 | 0.429 |  | EVPL | -0.406 |
| RP11-159H10.3 | 0.535 | NAA16 | 0.466 | KMT2C | 0.429 |  | RP11-380M21.2 | -0.406 |
| RBM12B | 0.535 | C18orf8 | 0.466 | WHSC1L1 | 0.429 |  | ADAMTSL5 | -0.406 |
| PHYHIPL | 0.535 | NOVA2 | 0.466 | CCDC3 | 0.429 |  | ZC3H12A | -0.407 |
| TXNDC16 | 0.535 | PNMAL1 | 0.466 | TADA2A | 0.429 |  | ALS2CL | -0.407 |
| ZNF473 | 0.535 | MARCKSL1 | 0.465 | KAT7 | 0.429 |  | NFKBIZ | -0.407 |
| RAB33A | 0.535 | KIAA0895 | 0.465 | LINC00630 | 0.429 |  | LRRC8E | -0.407 |
| FNDC5 | 0.534 | RSBN1L | 0.465 | HECW2 | 0.428 |  | NPTN | -0.408 |
| ABI2 | 0.534 | MTMR9 | 0.465 | TRA2B | 0.428 |  | ITGA3 | -0.408 |
| MED12L | 0.534 | TRMT10B | 0.465 | EFHC1 | 0.428 |  | KRT18P11 | -0.408 |
| TRIM36 | 0.534 | APBB1 | 0.465 | REPS1 | 0.428 |  | RNF181 | -0.409 |
| SRSF1 | 0.534 | DTX1 | 0.465 | AC004893.11 | 0.428 |  | UBTD1 | -0.409 |
| RBBP8 | 0.534 | RAB2B | 0.465 | RP11-155G14.5 | 0.428 |  | OBFC1 | -0.409 |
| AC006547.14 | 0.534 | ANKRD13B | 0.465 | AP3M2 | 0.428 |  | CTSC | -0.409 |
| RGAG4 | 0.534 | SOCS7 | 0.465 | MMP16 | 0.428 |  | IL18 | -0.409 |
| BEX1 | 0.534 | DTNA | 0.465 | KDELC1 | 0.428 |  | KRT7 | -0.409 |
| GDI1 | 0.534 | ZCCHC11 | 0.464 | EFS | 0.428 |  | SLC16A3 | -0.409 |
| RP11-797H7.1 | 0.533 | CCDC39 | 0.464 | TMED8 | 0.428 |  | ACSS2 | -0.409 |
| APC2 | 0.533 | N4BP2 | 0.464 | FANCA | 0.428 |  | AQP3 | -0.41 |
| USP48 | 0.532 | CDKN2AIP | 0.464 | ZNF780B | 0.428 |  | SLC25A43 | -0.41 |
| TTLL7 | 0.532 | FAR1 | 0.464 | FUZ | 0.428 |  | SGMS1 | -0.411 |
| AC016725.4 | 0.532 | SCN3B | 0.464 | ZFP69B | 0.427 |  | KCNN4 | -0.411 |
| DCAF16 | 0.532 | SEMA6D | 0.464 | MCM6 | 0.427 |  | HLA-C | -0.412 |
| SMAD4 | 0.532 | POLI | 0.464 | NLGN1 | 0.427 |  | AC093673.5 | -0.412 |
| XKR7 | 0.532 | LRCH2 | 0.464 | YTHDC1 | 0.427 |  | FAM129B | -0.412 |
| SLC16A10 | 0.531 | CDK5R2 | 0.463 | RP11-127B20.3 | 0.427 |  | RP11-783K16.5 | -0.412 |
| DCX | 0.531 | CDC25A | 0.463 | MDN1 | 0.427 |  | B2M | -0.412 |
| RBMX | 0.531 | SEMA6A | 0.463 | FUT9 | 0.427 |  | RP13-890H12.2 | -0.412 |
| TEKT2 | 0.53 | CNOT7 | 0.463 | BAZ1B | 0.427 |  | PVRL2 | -0.412 |
| SYT14 | 0.53 | TIMELESS | 0.463 | SENP1 | 0.427 |  | CD151 | -0.413 |
| ZNF496 | 0.53 | BIVM | 0.463 | SYT1 | 0.427 |  | TAGLN2 | -0.414 |
| CHN1 | 0.53 | KANSL1 | 0.463 | SERP2 | 0.427 |  | RBKS | -0.414 |
| HES6 | 0.53 | NOL9 | 0.462 | RPAIN | 0.427 |  | RAB43 | -0.414 |
| C6orf3 | 0.53 | SSX2IP | 0.462 | PLEKHJ1 | 0.427 |  | PLCD3 | -0.414 |
| INSM2 | 0.53 | RP11-504P24.3 | 0.462 | LINC00339 | 0.426 |  | CLU | -0.415 |
| FSD1 | 0.53 | CTD-2314G24.2 | 0.462 | AL161915.1 | 0.426 |  | RXRA | -0.415 |
| BSN | 0.529 | RNF182 | 0.462 | MDM4 | 0.426 |  | ABCC2 | -0.415 |
| BHLHE22 | 0.529 | ZNF292 | 0.462 | RANBP2 | 0.426 |  | NTN4 | -0.415 |
| CEND1 | 0.529 | SMIM8 | 0.462 | CCDC112 | 0.426 |  | ALDOA | -0.415 |
| PAPSS1 | 0.528 | ARID1B | 0.462 | DGKB | 0.426 |  | ETHE1 | -0.415 |
| WASF1 | 0.528 | UHRF2 | 0.462 | IKBKAP | 0.426 |  | RP11-157P1.4 | -0.415 |
| CHD7 | 0.528 | ZFHX2 | 0.462 | ING4 | 0.426 |  | GHITM | -0.416 |
| PAX5 | 0.528 | ZNF764 | 0.462 | KATNAL1 | 0.426 |  | OSGIN1 | -0.416 |
| SLC4A8 | 0.528 | CENPV | 0.462 | PPM1A | 0.426 |  | KRT18P38 | -0.417 |
| WNK3 | 0.528 | CEP192 | 0.462 | CHRNA3 | 0.426 |  | TAPBP | -0.417 |
| NRSN1 | 0.527 | ZNF227 | 0.462 | ST8SIA2 | 0.426 |  | RP11-395B7.7 | -0.417 |
| FYN | 0.527 | ZNF417 | 0.462 | CBX2 | 0.426 |  | ZYX | -0.417 |
| NECAB1 | 0.527 | SRRM1 | 0.461 | RP11-13N13.2 | 0.426 |  | RAB27B | -0.417 |
| FAM76B | 0.527 | ZBTB8A | 0.461 | PANK4 | 0.425 |  | PTK6 | -0.417 |
| ZFP1 | 0.527 | C1orf111 | 0.461 | ITGB3BP | 0.425 |  | MT-CO2 | -0.417 |
| EMILIN3 | 0.527 | CTNNA2 | 0.461 | H3F3A | 0.425 |  | ACAA1 | -0.418 |
| TCEAL2 | 0.527 | SDAD1 | 0.461 | RMND5A | 0.425 |  | MAFK | -0.418 |
| ATP2B3 | 0.527 | ZNF713 | 0.461 | ZNF197 | 0.425 |  | MAP3K8 | -0.418 |
| CNOT6L | 0.526 | LRRC10B | 0.461 | ACPL2 | 0.425 |  | PAPSS2 | -0.418 |
| C11orf95 | 0.526 | DDX51 | 0.461 | EXOSC9 | 0.425 |  | TMEM92 | -0.418 |
| RP11-540B6.3 | 0.526 | MGA | 0.461 | ZBTB12 | 0.425 |  | VDAC1P1 | -0.418 |
| MED14 | 0.526 | BTBD17 | 0.461 | RP11-262H14.1 | 0.425 |  | ANXA2P1 | -0.419 |
| UBQLN2 | 0.526 | PIN1 | 0.461 | PTCH1 | 0.425 |  | GALNT10 | -0.419 |
| TMEM201 | 0.525 | SEZ6L | 0.461 | ZNF189 | 0.425 |  | MYL12A | -0.419 |
| KIF2A | 0.525 | PRDM2 | 0.46 | GTF3C4 | 0.425 |  | S100P | -0.42 |
| TAL2 | 0.525 | MCM2 | 0.46 | CCKBR | 0.425 |  | IER5L | -0.42 |
| CAMSAP1 | 0.525 | YEATS2-AS1 | 0.46 | POLD3 | 0.425 |  | P4HB | -0.42 |
| RP11-715J22.6 | 0.525 | ATAT1 | 0.46 | GNB3 | 0.425 |  | RP11-290F20.1 | -0.42 |
| SCML2 | 0.525 | KIAA1456 | 0.46 | PHC1 | 0.425 |  | ADAM15 | -0.421 |
| TOPBP1 | 0.524 | KCNB2 | 0.46 | TUBGCP4 | 0.425 |  | CD55 | -0.421 |
| C4orf46 | 0.524 | RP11-408A13.4 | 0.46 | RTTN | 0.425 |  | HES1 | -0.421 |
| EML5 | 0.524 | CNTFR | 0.46 | CDKN2D | 0.425 |  | P4HA2 | -0.421 |
| ZNF81 | 0.524 | MAPK8IP1 | 0.46 | GHRH | 0.425 |  | CLTB | -0.421 |
| UPF3B | 0.524 | KMT2A | 0.46 | SYNJ1 | 0.425 |  | NANS | -0.421 |
| AGO3 | 0.523 | TMEM179 | 0.46 | KLHL13 | 0.425 |  | C10orf54 | -0.421 |
| SCAI | 0.523 | NPIPA1 | 0.46 | MASP2 | 0.424 |  | ADM | -0.421 |
| AKAP5 | 0.523 | NUDT21 | 0.46 | AC096677.1 | 0.424 |  | TM9SF1 | -0.421 |
| SNRPD1 | 0.523 | BZRAP1 | 0.46 | FLVCR1 | 0.424 |  | FAH | -0.421 |
| PDXP | 0.523 | ZNF708 | 0.46 | AC092811.1 | 0.424 |  | FAM114A1 | -0.422 |
| EML6 | 0.522 | SFI1 | 0.46 | ITGA9 | 0.424 |  | HEXB | -0.422 |
| RP11-1415C14.4 | 0.522 | CCDC28B | 0.459 | PRKAR2B | 0.424 |  | VDAC1 | -0.422 |
| RP11-815I9.4 | 0.522 | FOXD2-AS1 | 0.459 | ZNF775 | 0.424 |  | TAP1 | -0.422 |
| SUGP2 | 0.522 | SRP9 | 0.459 | SFRP5 | 0.424 |  | UPP1 | -0.422 |
| UNC80 | 0.521 | ELOVL2 | 0.459 | SVIP | 0.424 |  | TSKU | -0.422 |
| CLSTN2 | 0.521 | RMI1 | 0.459 | RAD51AP1 | 0.424 |  | SEMA4B | -0.422 |
| FZD3 | 0.521 | ZNF782 | 0.459 | RP11-551L14.1 | 0.424 |  | BCAR3 | -0.423 |
| ENHO | 0.521 | MIR600HG | 0.459 | SNN | 0.424 |  | CASP4 | -0.423 |
| RRM1 | 0.521 | USP31 | 0.459 | TTC25 | 0.424 |  | FLVCR2 | -0.423 |
| SCG3 | 0.521 | HMGXB4 | 0.459 | ZNF521 | 0.424 |  | PIGB | -0.423 |
| ZNF709 | 0.521 | S100PBP | 0.458 | MAPRE2 | 0.424 |  | PADI1 | -0.424 |
| SNAPC3 | 0.52 | SGTB | 0.458 | SETBP1 | 0.424 |  | EGFL7 | -0.424 |
| BRSK2 | 0.52 | XPO5 | 0.458 | CTB-31O20.2 | 0.424 |  | AGPAT2 | -0.425 |
| ZNF397 | 0.52 | ADCY1 | 0.458 | RTN2 | 0.424 |  | SIAE | -0.425 |
| MAD2L2 | 0.519 | KIAA1324L | 0.458 | NUP62 | 0.424 |  | LINC00920 | -0.425 |
| USP1 | 0.519 | PIDD | 0.458 | SS18L1 | 0.424 |  | ACSF2 | -0.425 |
| BCLAF1 | 0.519 | NPAT | 0.458 | FAM122C | 0.424 |  | SH2D3A | -0.425 |
| NRF1 | 0.519 | PRPH | 0.458 | RP11-1114A5.5 | 0.424 |  | KRT8P10 | -0.426 |
| WEE1 | 0.519 | SHF | 0.458 | TCEANC2 | 0.423 |  | TRIM16 | -0.426 |
| MZT1 | 0.519 | ZNF286A | 0.458 | OSBPL6 | 0.423 |  | SLPI | -0.426 |
| ZNF236 | 0.519 | ESCO1 | 0.458 | CELSR3 | 0.423 |  | C1QTNF6 | -0.426 |
| ZNF324B | 0.519 | SAFB | 0.458 | U2SURP | 0.423 |  | EHBP1L1 | -0.427 |
| HCFC1 | 0.519 | ZNF266 | 0.458 | RP3-525N10.2 | 0.423 |  | HADHB | -0.428 |
| TARDBP | 0.518 | ZNF674-AS1 | 0.458 | PTPRZ1 | 0.423 |  | OSMR | -0.428 |
| IPO9 | 0.518 | RP3-327A19.5 | 0.458 | ST18 | 0.423 |  | HIBADH | -0.428 |
| AGPAT4 | 0.518 | CXCR4 | 0.457 | GAB2 | 0.423 |  | IL15RA | -0.428 |
| ZNF483 | 0.518 | ZNF852 | 0.457 | GPR162 | 0.423 |  | KIAA1217 | -0.428 |
| RP11-111M22.4 | 0.518 | HMGB2 | 0.457 | C14orf23 | 0.423 |  | SRXN1 | -0.428 |
| RP4-773A18.4 | 0.517 | MTX3 | 0.457 | FAN1 | 0.423 |  | LPP | -0.429 |
| FAM161A | 0.517 | BRAF | 0.457 | SCAPER | 0.423 |  | TRIM38 | -0.429 |
| RNGTT | 0.517 | WRN | 0.457 | GINS2 | 0.423 |  | CLIC1 | -0.429 |
| CELF1 | 0.517 | KIAA2026 | 0.457 | RP11-400F19.18 | 0.423 |  | MICALL2 | -0.429 |
| SOS1-IT1 | 0.516 | ELAVL2 | 0.457 | VEZF1 | 0.423 |  | KRT8P3 | -0.429 |
| SEPT11 | 0.516 | ZIC5 | 0.457 | MPND | 0.423 |  | EFR3A | -0.429 |
| FBLL1 | 0.516 | CTC-425O23.2 | 0.457 | RP11-15H20.5 | 0.423 |  | SURF4 | -0.429 |
| MRAP2 | 0.516 | CPT1C | 0.457 | MED14-AS1 | 0.423 |  | CLCF1 | -0.429 |
| CCDC136 | 0.516 | RIMS4 | 0.457 | MECP2 | 0.423 |  | TMEM159 | -0.429 |
| ZNF250 | 0.516 | GABPA | 0.457 | CCDC74A | 0.422 |  | TGFBR2 | -0.43 |
| RCOR2 | 0.516 | ZBTB18 | 0.456 | LRCH3 | 0.422 |  | SNAP23 | -0.43 |
| TTBK2 | 0.516 | DLX5 | 0.456 | SGCB | 0.422 |  | TAP2 | -0.431 |
| MBTD1 | 0.516 | CSRNP2 | 0.456 | DCP2 | 0.422 |  | OSTF1 | -0.431 |
| WDR7 | 0.516 | TEX9 | 0.456 | ARMC2 | 0.422 |  | EEF1DP1 | -0.431 |
| DRAXIN | 0.515 | RP11-156E6.1 | 0.456 | CEP41 | 0.422 |  | TGFBI | -0.432 |
| HPCA | 0.515 | DGKE | 0.456 | CTD-2647L4.4 | 0.422 |  | AHR | -0.432 |
| SLAIN1 | 0.515 | ZSCAN30 | 0.456 | PENK | 0.422 |  | FURIN | -0.432 |
| LRRC49 | 0.515 | RP11-206L10.11 | 0.455 | GAD2 | 0.422 |  | TLCD2 | -0.432 |
| HMGN2P3 | 0.515 | CDK19 | 0.455 | UBR7 | 0.422 |  | RHOD | -0.433 |
| MYT1 | 0.515 | HECA | 0.455 | FAM64A | 0.422 |  | RP11-96H19.1 | -0.433 |
| PTCHD2 | 0.514 | WTAP | 0.455 | ZBTB14 | 0.422 |  | FXYD5 | -0.433 |
| RBBP4 | 0.514 | RALYL | 0.455 | ME2 | 0.422 |  | ERGIC1 | -0.434 |
| ZNF124 | 0.514 | RP11-122A3.2 | 0.455 | ZGLP1 | 0.422 |  | TCIRG1 | -0.434 |
| C3orf70 | 0.514 | RP11-958N24.1 | 0.455 | ZFP30 | 0.422 |  | MOB3C | -0.435 |
| KIF3A | 0.514 | RP11-1055B8.6 | 0.455 | ATP1A3 | 0.422 |  | RP11-285F7.2 | -0.435 |
| DUSP26 | 0.514 | DONSON | 0.455 | ZNF446 | 0.422 |  | TMBIM6 | -0.435 |
| BCL7A | 0.514 | RP3-508I15.9 | 0.455 | E2F1 | 0.422 |  | CD68 | -0.435 |
| RP11-95O2.5 | 0.514 | ZBTB40 | 0.454 | C21orf91 | 0.422 |  | MISP | -0.435 |
| ILF3 | 0.514 | PUM1 | 0.454 | SRRD | 0.422 |  | REEP6 | -0.435 |
| CCM2L | 0.514 | ADD2 | 0.454 | FAM19A5 | 0.422 |  | RBM47 | -0.436 |
| CASP8AP2 | 0.513 | CCNT2 | 0.454 | BHLHB9 | 0.422 |  | CRIM1 | -0.437 |
| GPC2 | 0.513 | PDZRN3 | 0.454 | RP1-228H13.5 | 0.421 |  | TKT | -0.437 |
| RAMP2 | 0.513 | JAKMIP1 | 0.454 | DLX2 | 0.421 |  | CD63 | -0.437 |
| SRRM4 | 0.512 | LATS1 | 0.454 | KCNMB2 | 0.421 |  | NPC2 | -0.437 |
| FAM171A2 | 0.512 | MYEF2 | 0.454 | CXXC4 | 0.421 |  | EPHB4 | -0.438 |
| TRBV26OR9-2 | 0.511 | TCF12 | 0.454 | SOBP | 0.421 |  | IFITM3 | -0.438 |
| C2orf44 | 0.51 | BCL2 | 0.454 | RP11-513M16.8 | 0.421 |  | SH2D4A | -0.439 |
| LOXL3 | 0.51 | SPTBN4 | 0.454 | RP11-573M3.3 | 0.421 |  | LIPA | -0.439 |
| KCNH8 | 0.51 | AC007193.9 | 0.454 | CHD4 | 0.421 |  | CHP1 | -0.439 |
| STXBP5L | 0.51 | PLA2G3 | 0.454 | SMARCD1 | 0.421 |  | S100A6 | -0.44 |
| RBPJ | 0.51 | TMEM178A | 0.453 | NDRG4 | 0.421 |  | LIPH | -0.44 |
| AGTPBP1 | 0.51 | KLHDC3 | 0.453 | RP11-635N19.1 | 0.421 |  | PRR13 | -0.441 |
| RIC8B | 0.51 | THAP5 | 0.453 | SHC2 | 0.421 |  | SMAD3 | -0.441 |
| ZNF286B | 0.51 | DENND2A | 0.453 | ZNF627 | 0.421 |  | LMF1 | -0.441 |
| KIF1B | 0.509 | BRD3 | 0.453 | ZNF490 | 0.421 |  | TSPO | -0.441 |
| KDM1A | 0.509 | NCR3LG1 | 0.453 | AC069278.4 | 0.421 |  | KRT8P45 | -0.442 |
| WDR47 | 0.509 | REC8 | 0.453 | HIRA | 0.421 |  | ACY1 | -0.442 |
| PLEKHO1 | 0.509 | SMPD3 | 0.453 | NAP1L3 | 0.421 |  | SP100 | -0.443 |
| AGPAT5 | 0.509 | TSHZ1 | 0.453 | RP11-1114A5.4 | 0.421 |  | NT5E | -0.443 |
| TAF5 | 0.509 | MIR7-3HG | 0.453 | SLC35E2 | 0.42 |  | GSTK1 | -0.443 |
| TTL | 0.508 | EIF4ENIF1 | 0.453 | MTR | 0.42 |  | ADAM9 | -0.443 |
| RP11-395I6.3 | 0.508 | RPRM | 0.452 | SMARCC1 | 0.42 |  | SPRYD3 | -0.444 |
| GPRIN1 | 0.508 | RP11-798M19.6 | 0.452 | LSAMP | 0.42 |  | PYGL | -0.444 |
| SYNGAP1 | 0.508 | KHDRBS3 | 0.452 | CTD-2089N3.2 | 0.42 |  | CASP8 | -0.445 |
| TUBGCP3 | 0.508 | MPDZ | 0.452 | SNCAIP | 0.42 |  | WWTR1 | -0.445 |
| PCNT | 0.508 | CCDC15 | 0.452 | EBF1 | 0.42 |  | SLC8B1 | -0.445 |
| RPA2 | 0.507 | NCAPD3 | 0.452 | ZNF322 | 0.42 |  | ERGIC3 | -0.445 |
| SYT11 | 0.507 | PPFIA2 | 0.452 | RRAGD | 0.42 |  | PPIC | -0.446 |
| ICA1L | 0.507 | NBEA | 0.452 | FAM229B | 0.42 |  | MFSD5 | -0.446 |
| TUBB2B | 0.507 | ABHD13 | 0.452 | CCAR2 | 0.42 |  | AJUBA | -0.446 |
| PRR3 | 0.507 | MBD1 | 0.452 | RP11-347C18.5 | 0.42 |  | SYPL1 | -0.447 |
| GNG2 | 0.507 | ZNF71 | 0.452 | DIRAS2 | 0.42 |  | LTBR | -0.447 |
| HAUS1 | 0.507 | TP73 | 0.451 | FANCC | 0.42 |  | H2AFJ | -0.447 |
| ZNF532 | 0.507 | PHF13 | 0.451 | SCN8A | 0.42 |  | KRT18 | -0.447 |
| NAPB | 0.507 | PPP1R8 | 0.451 | DGKH | 0.42 |  | SMOX | -0.447 |
| DLGAP3 | 0.506 | AGAP1 | 0.451 | LRFN5 | 0.42 |  | ELOVL1 | -0.448 |
| KIAA1009 | 0.506 | MSL2 | 0.451 | HSDL1 | 0.42 |  | TM4SF1 | -0.448 |
| RP11-732M18.3 | 0.506 | AUTS2 | 0.451 | CTD-2286N8.2 | 0.42 |  | KRT80 | -0.449 |
| LZTS1 | 0.506 | QSER1 | 0.451 | AMER3 | 0.419 |  | B3GNT3 | -0.449 |
| RP11-819C21.1 | 0.506 | STX2 | 0.451 | CCDC14 | 0.419 |  | RRAS | -0.449 |
| RNF38 | 0.505 | GNAO1 | 0.451 | GRK4 | 0.419 |  | BCL2L12 | -0.449 |
| ZNF195 | 0.505 | LINC00909 | 0.451 | RP11-83A24.2 | 0.419 |  | RETSAT | -0.45 |
| PTPRO | 0.505 | ZNF182 | 0.451 | SNCB | 0.419 |  | KCTD11 | -0.45 |
| CTDSPL2 | 0.505 | RCC2 | 0.45 | SMC3 | 0.419 |  | PPAP2C | -0.45 |
| RFX7 | 0.505 | VANGL2 | 0.45 | KNTC1 | 0.419 |  | GPR108 | -0.45 |
| KLHL11 | 0.505 | IVNS1ABP | 0.45 | CDH24 | 0.419 |  | RAB20 | -0.451 |
| TRIM37 | 0.505 | RP11-391M1.4 | 0.45 | CRABP1 | 0.419 |  | MVP | -0.451 |
| ZNF543 | 0.505 | BBS7 | 0.45 | BLM | 0.419 |  | SPATA20 | -0.451 |
| CTD-2619J13.17 | 0.505 | FANCF | 0.45 | ZFP90 | 0.419 |  | KDELR3 | -0.451 |
| ZNF512 | 0.504 | AP001877.1 | 0.45 | APPBP2 | 0.419 |  | REST | -0.452 |
| RP11-384P7.7 | 0.504 | ATP8A2 | 0.45 | SLC25A19 | 0.419 |  | MICA | -0.452 |
| FRRS1L | 0.504 | GTF2A1 | 0.45 | AC005003.1 | 0.419 |  | PPIB | -0.452 |
| AZI1 | 0.504 | AC116407.2 | 0.45 | NCAPH2 | 0.419 |  | RP5-1142A6.9 | -0.452 |
| ZNF557 | 0.504 | RP11-742D12.2 | 0.45 | SMPD4 | 0.418 |  | MIR24-2 | -0.452 |
| BCOR | 0.504 | ZNF132 | 0.45 | PMS1 | 0.418 |  | SAT1 | -0.452 |
| TCEAL5 | 0.504 | TIAM1 | 0.45 | MED28 | 0.418 |  | PRSS23 | -0.453 |
| ZMYM4 | 0.503 | CHAF1B | 0.45 | TMEM170B | 0.418 |  | ANXA2 | -0.453 |
| TMEM81 | 0.503 | RRP1B | 0.45 | NYAP1 | 0.418 |  | CDC42EP1 | -0.454 |
| MAATS1 | 0.503 | TUG1 | 0.45 | ARL14EP | 0.418 |  | FNDC3B | -0.455 |
| ARL10 | 0.503 | FAM104B | 0.45 | FAM60A | 0.418 |  | SERPINB1 | -0.455 |
| CDKAL1 | 0.503 | SV2A | 0.449 | PLXNC1 | 0.418 |  | CLIP1 | -0.455 |
| STXBP1 | 0.503 | CNRIP1 | 0.449 | RIMBP2 | 0.418 |  | CD99 | -0.455 |
| ATCAY | 0.503 | DHX36 | 0.449 | C19orf57 | 0.418 |  | SLC22A18 | -0.456 |
| FANCB | 0.503 | MYB | 0.449 | ZFP14 | 0.418 |  | NEAT1 | -0.456 |
| RSBN1 | 0.502 | AC004987.9 | 0.449 | MAPK8IP2 | 0.418 |  | SDSL | -0.456 |
| KLHDC8A | 0.502 | ZNF273 | 0.449 | CCDC138 | 0.417 |  | KIFC3 | -0.456 |
| ZNF678 | 0.502 | GDAP1 | 0.449 | ZKSCAN7 | 0.417 |  | ITGB4 | -0.456 |
| MAD2L1 | 0.502 | STK33 | 0.449 | PEX5L | 0.417 |  | TRIP10 | -0.456 |
| CCNE2 | 0.502 | FEN1 | 0.449 | TET2 | 0.417 |  | SERTAD1 | -0.456 |
| LINC00537 | 0.502 | RP11-214K3.21 | 0.449 | SRSF3 | 0.417 |  | TES | -0.458 |
| CPSF6 | 0.502 | SOX1 | 0.449 | SESN1 | 0.417 |  | IL4R | -0.458 |
| GPR137C | 0.502 | WSCD1 | 0.449 | TBP | 0.417 |  | NQO1 | -0.458 |
| HDX | 0.502 | ROCK1 | 0.449 | RN7SL481P | 0.417 |  | KIF1C | -0.458 |
| RAD54L | 0.501 | DOK6 | 0.449 | FAM131B | 0.417 |  | SLC39A1 | -0.459 |
| CAMKV | 0.501 | ZNF17 | 0.449 | FAM92A1 | 0.417 |  | SUMF1 | -0.459 |
| PCDHGC4 | 0.501 | SPECC1L | 0.449 | RP11-137H2.6 | 0.417 |  | ZFP36L1 | -0.459 |
| BAI3 | 0.501 | GTPBP1 | 0.449 | CHAMP1 | 0.417 |  | SUCLG2 | -0.461 |
| IGSF9B | 0.501 | BCORL1 | 0.449 | RBFOX1 | 0.417 |  | SQSTM1 | -0.461 |
| DACH1 | 0.501 | SLC25A33 | 0.448 | ZNF264 | 0.417 |  | EGFR | -0.461 |
| NOL4 | 0.501 | RLF | 0.448 | CHD6 | 0.417 |  | ITPRIPL2 | -0.461 |
| SYP | 0.501 | TROVE2 | 0.448 | RPGR | 0.417 |  | TMEM214 | -0.462 |
| AC073415.2 | 0.5 | PCBP4 | 0.448 | FGD1 | 0.417 |  | SSH3 | -0.462 |
| AC093838.4 | 0.5 | LIN54 | 0.448 | BEX4 | 0.417 |  | ARHGAP27 | -0.462 |
| TMEM169 | 0.5 | TUBB2BP1 | 0.448 | KIF3C | 0.416 |  | LINC00963 | -0.463 |
| VWA5B2 | 0.5 | ZNF138 | 0.448 | RP11-646I6.5 | 0.416 |  | CAPN2 | -0.464 |
| RP11-1415C14.3 | 0.5 | TRIM24 | 0.448 | MAPK10 | 0.416 |  | SLC16A5 | -0.464 |
| TIGD3 | 0.5 | FAM27E3 | 0.448 | HMGN2P4 | 0.416 |  | SNX33 | -0.465 |
| ATAD5 | 0.5 | ZBTB44 | 0.448 | EHMT2 | 0.416 |  | ORAI3 | -0.465 |
| CLSPN | 0.499 | KCNA6 | 0.448 | CBX5 | 0.416 |  | LAMA5 | -0.466 |
| FAXC | 0.499 | COL2A1 | 0.448 | FAM222A | 0.416 |  | EPHA2 | -0.468 |
| ZBTB39 | 0.499 | C12orf65 | 0.448 | PCDH8 | 0.416 |  | SLC35D2 | -0.468 |
| C14orf132 | 0.499 | LINC00404 | 0.448 | NRXN3 | 0.416 |  | GPRC5A | -0.469 |
| C17orf80 | 0.499 | PKD1P6 | 0.448 | PKD1 | 0.416 |  | RNF135 | -0.469 |
| RP11-258C19.7 | 0.499 | AC004381.6 | 0.448 | LRRC37B | 0.416 |  | NPAS2 | -0.47 |
| PHOSPHO2 | 0.498 | RNF157 | 0.448 | THOC1 | 0.416 |  | VAMP8 | -0.471 |
| RP11-242F4.2 | 0.498 | CEP76 | 0.448 | NETO1 | 0.416 |  | GLB1 | -0.472 |
| SARM1 | 0.498 | ZNF460 | 0.448 | CELF5 | 0.416 |  | TOMM7 | -0.472 |
| PAXBP1-AS1 | 0.498 | DHX9 | 0.447 | TTC28 | 0.416 |  | ANXA1 | -0.472 |
| E2F2 | 0.497 | PROX1 | 0.447 | CCDC157 | 0.416 |  | GADD45B | -0.473 |
| PITHD1 | 0.497 | ISL1 | 0.447 | KDM6A | 0.416 |  | CCND1 | -0.475 |
| GPX7 | 0.497 | POU3F2 | 0.447 | GRIPAP1 | 0.416 |  | CEBPB | -0.477 |
| EPC2 | 0.497 | PVRIG | 0.447 | RLIM | 0.416 |  | LAMB2 | -0.478 |
| EZH2 | 0.497 | C1QL3 | 0.447 | ATG4C | 0.415 |  | NQO2 | -0.478 |
| JAM3 | 0.497 | CCDC34 | 0.447 | ROBO2 | 0.415 |  | NCOA4 | -0.478 |
| KIAA1328 | 0.497 | TRIM13 | 0.447 | SENP5 | 0.415 |  | EDEM2 | -0.478 |
| SHISA7 | 0.497 | RP11-966I7.2 | 0.447 | UBE2K | 0.415 |  | S100A16 | -0.479 |
| USP11 | 0.497 | RP11-894P9.1 | 0.447 | THAP6 | 0.415 |  | UNC93B1 | -0.479 |
| RP11-258C19.5 | 0.497 | HERC2 | 0.447 | RIMS2 | 0.415 |  | TMED3 | -0.479 |
| AGO1 | 0.496 | LUC7L | 0.447 | KIF24 | 0.415 |  | TMEM63A | -0.48 |
| TMEM206 | 0.496 | RASL10B | 0.447 | SOX5 | 0.415 |  | MIR4435-1HG | -0.48 |
| GRM4 | 0.496 | DCAF7 | 0.447 | PXMP2 | 0.415 |  | ANXA2P2 | -0.48 |
| SERAC1 | 0.496 | LINC00526 | 0.447 | NFATC3 | 0.415 |  | SIL1 | -0.481 |
| RP1-266L20.2 | 0.496 | KIAA2022 | 0.447 | KRBA2 | 0.415 |  | B4GALT1 | -0.482 |
| GLT1D1 | 0.496 | PDIK1L | 0.446 | PGS1 | 0.415 |  | MLKL | -0.482 |
| SUZ12P | 0.496 | IQCC | 0.446 | UBA2 | 0.415 |  | TCF7L2 | -0.483 |
| TTC9B | 0.496 | ATXN7L2 | 0.446 | MCM8 | 0.415 |  | SH3D19 | -0.484 |
| CECR2 | 0.496 | SEMA6C | 0.446 | SLC32A1 | 0.415 |  | RHOC | -0.486 |
| HPS4 | 0.496 | REEP1 | 0.446 | CNTN4 | 0.414 |  | LINC00152 | -0.486 |
| ZBED4 | 0.496 | LARP1B | 0.446 | MAP6D1 | 0.414 |  | PLD1 | -0.488 |
| CADPS | 0.495 | RP11-1023L17.1 | 0.446 | RAB28 | 0.414 |  | METRNL | -0.488 |
| RP11-396K3.1 | 0.495 | PGBD1 | 0.446 | RP11-1C8.4 | 0.414 |  | EPAS1 | -0.489 |
| ZNF510 | 0.495 | MAGI2 | 0.446 | TET1 | 0.414 |  | TNFRSF10B | -0.489 |
| PSD | 0.495 | PIPOX | 0.446 | KCNA1 | 0.414 |  | SDC4 | -0.489 |
| POLA2 | 0.495 | ZNF271 | 0.446 | LIG4 | 0.414 |  | BHLHE40 | -0.49 |
| NTN3 | 0.495 | DNMT1 | 0.446 | KIAA1737 | 0.414 |  | SQRDL | -0.491 |
| PARD6G | 0.495 | CTD-3138B18.6 | 0.446 | RP11-73C9.1 | 0.414 |  | DHX32 | -0.492 |
| DOT1L | 0.495 | ZRANB2 | 0.445 | EFNB3 | 0.414 |  | ATP6V0E1 | -0.493 |
| PDZD4 | 0.495 | NEUROG2 | 0.445 | ZNF230 | 0.414 |  | TGIF1 | -0.493 |
| SSBP3 | 0.494 | ELMOD2 | 0.445 | ZNF616 | 0.414 |  | TMCO4 | -0.494 |
| PDE7A | 0.494 | ZBTB24 | 0.445 | BRD1 | 0.414 |  | ARPC1B | -0.494 |
| CSTF3 | 0.494 | KPNA5 | 0.445 | STAG2 | 0.414 |  | MIR22HG | -0.494 |
| FOXO3B | 0.494 | RADIL | 0.445 | HTATSF1 | 0.414 |  | C19orf33 | -0.494 |
| UNK | 0.494 | DLX6-AS1 | 0.445 | DNALI1 | 0.413 |  | PLEC | -0.495 |
| TNPO2 | 0.494 | ASRGL1 | 0.445 | FAM72B | 0.413 |  | AHNAK | -0.495 |
| ARID1A | 0.493 | MEGF11 | 0.445 | KCNK2 | 0.413 |  | TNFRSF1A | -0.496 |
| SFPQ | 0.493 | ZNF559 | 0.445 | AC117395.1 | 0.413 |  | ZFP36 | -0.497 |
| TTC21B | 0.493 | ZNF334 | 0.445 | CYP2U1 | 0.413 |  | CTSD | -0.498 |
| H1FX-AS1 | 0.493 | YTHDF2 | 0.444 | PRUNE2 | 0.413 |  | PTGES | -0.499 |
| TBC1D24 | 0.493 | RGS16 | 0.444 | AC005329.7 | 0.413 |  | HLA-E | -0.501 |
| UBTF | 0.493 | AC096772.6 | 0.444 | DZANK1 | 0.413 |  | YAP1 | -0.501 |
| CRHR1-IT1 | 0.493 | RP11-16N11.2 | 0.444 | NONO | 0.413 |  | TMED9 | -0.502 |
| CHAF1A | 0.493 | FBXO45 | 0.444 | RP1-161N10.1 | 0.413 |  | ABCC3 | -0.504 |
| PLCG1 | 0.493 | RP3-428L16.2 | 0.444 | FMR1 | 0.413 |  | TRPM4 | -0.504 |
| KHDRBS1 | 0.492 | CNTNAP2 | 0.444 | RP1-39G22.7 | 0.412 |  | S100A11 | -0.505 |
| CHRNB2 | 0.492 | MADD | 0.444 | RP11-656D10.6 | 0.412 |  | AVPI1 | -0.505 |
| KIF1A | 0.492 | SF1 | 0.444 | RP5-994D16.9 | 0.412 |  | BAG3 | -0.505 |
| PTPRD | 0.492 | RP11-283I3.6 | 0.444 | POGK | 0.412 |  | LGALS3 | -0.505 |
| FGF14-AS2 | 0.492 | SLMO1 | 0.444 | TRIM67 | 0.412 |  | TRADD | -0.506 |
| VASH1 | 0.492 | HDHD2 | 0.444 | TIA1 | 0.412 |  | LMAN2 | -0.507 |
| CEP85 | 0.491 | DLL3 | 0.444 | TSN | 0.412 |  | CNN2 | -0.508 |
| GRIK3 | 0.491 | AP1S2 | 0.444 | RP11-121C2.2 | 0.412 |  | CTSZ | -0.509 |
| GPATCH2 | 0.491 | INTS7 | 0.443 | HELQ | 0.412 |  | LASP1 | -0.516 |
| RAD54L2 | 0.491 | ANGEL2 | 0.443 | AHI1 | 0.412 |  | HFE | -0.517 |
| PPM1L | 0.491 | FOXN2 | 0.443 | ZNF37BP | 0.412 |  | PIEZO1 | -0.518 |
| BMP2K | 0.491 | PLEKHM3 | 0.443 | MEN1 | 0.412 |  | CAST | -0.519 |
| SMARCA5 | 0.491 | DENND6A | 0.443 | FAM168A | 0.412 |  | SERPINB6 | -0.519 |
| PHIP | 0.491 | AADAT | 0.443 | RP11-203M5.7 | 0.412 |  | BCL9L | -0.52 |
| TULP4 | 0.491 | CYFIP2 | 0.443 | LYSMD4 | 0.412 |  | CTSA | -0.52 |
| TNKS | 0.491 | RIC3 | 0.443 | TERF2IP | 0.412 |  | TNFRSF12A | -0.521 |
| MTMR7 | 0.491 | MPHOSPH9 | 0.443 | ZNF778 | 0.412 |  | CTSL | -0.523 |
| PKIA | 0.491 | SOCS4 | 0.443 | TLK2 | 0.412 |  | RRBP1 | -0.523 |
| RP11-262H14.5 | 0.491 | GABRB3 | 0.443 | RP11-143K11.1 | 0.412 |  | S100A10 | -0.524 |
| RP11-111M22.2 | 0.491 | RP11-358M11.3 | 0.443 | SDK2 | 0.412 |  | ANXA4 | -0.526 |
| BRIP1 | 0.491 | PIP4K2B | 0.443 | LTN1 | 0.412 |  | BCL3 | -0.527 |
| SHD | 0.491 | ZNF77 | 0.443 | AP001469.7 | 0.412 |  | MYOF | -0.529 |
| TSPY26P | 0.491 | TMEM145 | 0.443 | NBPF1 | 0.411 |  | ALDH3B1 | -0.53 |
| PPP1R16B | 0.491 | AC018755.1 | 0.443 | WDTC1 | 0.411 |  | RHBDF1 | -0.534 |
| CD200 | 0.49 | HDAC6 | 0.443 | RAPGEF4 | 0.411 |  | LINC00857 | -0.535 |
| RP1-152L7.5 | 0.49 | RPS6KA6 | 0.443 | B3GAT1 | 0.411 |  | CD97 | -0.535 |
| KIAA1958 | 0.49 | PGBD5 | 0.442 | SUZ12 | 0.411 |  | TMBIM1 | -0.538 |
| NRXN2 | 0.49 | ORC4 | 0.442 | STRADA | 0.411 |  | IER3 | -0.538 |
| C1orf95 | 0.489 | XRCC5 | 0.442 | KLHL14 | 0.411 |  | PLIN3 | -0.541 |
| POLR2D | 0.489 | TOP2B | 0.442 | MPPED1 | 0.411 |  | CIB1 | -0.552 |
| HNRNPA3 | 0.489 | CACNA2D1 | 0.442 | MDH1B | 0.41 |  | LRP10 | -0.559 |
| CEP78 | 0.489 | PTAR1 | 0.442 | LINC00693 | 0.41 |  | ANXA11 | -0.567 |
| TPGS2 | 0.489 | HMX2 | 0.442 | GLCCI1 | 0.41 |  | LMNA | -0.57 |
| ZNF749 | 0.489 | CPSF7 | 0.442 | ERCC6L2 | 0.41 |  | POLD4 | -0.575 |

Table S4. GO annotation of PAQR3 co-expressed genes

| Ontology | ID | Description | Count | pvalue | p. adjust |
| --- | --- | --- | --- | --- | --- |
| BP | GO:0006260 | DNA replication | 63 | 9.26E-15 | 4.98E-11 |
| BP | GO:0048667 | cell morphogenesis involved in neuron differentiation | 90 | 3.51E-12 | 9.43E-09 |
| BP | GO:0018205 | peptidyl-lysine modification | 73 | 1.06E-11 | 1.90E-08 |
| BP | GO:0016570 | histone modification | 76 | 1.35E-10 | 1.82E-07 |
| BP | GO:0007409 | axonogenesis | 72 | 2.34E-10 | 2.52E-07 |
| BP | GO:0016569 | covalent chromatin modification | 76 | 5.81E-10 | 5.21E-07 |
| BP | GO:0061564 | axon development | 76 | 9.97E-10 | 7.66E-07 |
| BP | GO:0099504 | synaptic vesicle cycle | 39 | 1.41E-09 | 8.81E-07 |
| BP | GO:0007269 | neurotransmitter secretion | 37 | 1.47E-09 | 8.81E-07 |
| BP | GO:0099643 | signal release from synapse | 37 | 1.80E-09 | 9.69E-07 |
| BP | GO:0000380 | alternative mRNA splicing, via spliceosome | 20 | 5.77E-09 | 2.82E-06 |
| BP | GO:0000226 | microtubule cytoskeleton organization | 75 | 2.08E-08 | 8.98E-06 |
| BP | GO:0006261 | DNA-dependent DNA replication | 32 | 2.34E-08 | 8.98E-06 |
| BP | GO:0016571 | histone methylation | 32 | 2.34E-08 | 8.98E-06 |
| BP | GO:0098693 | regulation of synaptic vesicle cycle | 28 | 3.41E-08 | 1.22E-05 |
| BP | GO:0034968 | histone lysine methylation | 28 | 6.54E-08 | 2.20E-05 |
| BP | GO:0000381 | regulation of alternative mRNA splicing, via spliceosome | 16 | 8.18E-08 | 2.58E-05 |
| BP | GO:0023061 | signal release | 70 | 8.65E-08 | 2.58E-05 |
| BP | GO:0043543 | protein acylation | 45 | 1.14E-07 | 3.23E-05 |
| BP | GO:0050684 | regulation of mRNA processing | 27 | 2.04E-07 | 5.49E-05 |
| BP | GO:0072331 | signal transduction by p53 class mediator | 48 | 2.94E-07 | 7.29E-05 |
| BP | GO:0097479 | synaptic vesicle localization | 31 | 2.98E-07 | 7.29E-05 |
| BP | GO:0099003 | vesicle-mediated transport in synapse | 36 | 3.66E-07 | 8.57E-05 |
| BP | GO:0050657 | nucleic acid transport | 36 | 4.91E-07 | 0.000105593 |
| BP | GO:0050658 | RNA transport | 36 | 4.91E-07 | 0.000105593 |
| BP | GO:0044786 | cell cycle DNA replication | 18 | 5.40E-07 | 0.000111724 |
| BP | GO:0048024 | regulation of mRNA splicing, via spliceosome | 21 | 6.04E-07 | 0.000120341 |
| BP | GO:0031109 | microtubule polymerization or depolymerization | 24 | 6.31E-07 | 0.000121299 |
| BP | GO:0030900 | forebrain development | 60 | 7.08E-07 | 0.000130677 |
| BP | GO:0018394 | peptidyl-lysine acetylation | 34 | 7.29E-07 | 0.000130677 |
| BP | GO:0051236 | establishment of RNA localization | 36 | 7.53E-07 | 0.000130677 |
| BP | GO:0022406 | membrane docking | 32 | 7.89E-07 | 0.000132459 |
| BP | GO:0018022 | peptidyl-lysine methylation | 28 | 8.12E-07 | 0.000132459 |
| BP | GO:0006473 | protein acetylation | 38 | 8.57E-07 | 0.000135633 |
| BP | GO:0006310 | DNA recombination | 44 | 1.14E-06 | 0.000175556 |
| BP | GO:0006475 | internal protein amino acid acetylation | 33 | 1.38E-06 | 0.000199644 |
| BP | GO:0097091 | synaptic vesicle clustering | 9 | 1.40E-06 | 0.000199644 |
| BP | GO:0051648 | vesicle localization | 47 | 1.41E-06 | 0.000199644 |
| BP | GO:0008380 | RNA splicing | 67 | 1.50E-06 | 0.000206489 |
| BP | GO:0070507 | regulation of microtubule cytoskeleton organization | 34 | 1.73E-06 | 0.000232404 |
| BP | GO:0007019 | microtubule depolymerization | 13 | 1.78E-06 | 0.000234188 |
| BP | GO:0140056 | organelle localization by membrane tethering | 30 | 1.83E-06 | 0.000234417 |
| BP | GO:0017156 | calcium ion regulated exocytosis | 28 | 1.91E-06 | 0.000238428 |
| BP | GO:0018393 | internal peptidyl-lysine acetylation | 32 | 1.95E-06 | 0.000238428 |
| BP | GO:0007411 | axon guidance | 39 | 2.17E-06 | 0.000259026 |
| BP | GO:1903311 | regulation of mRNA metabolic process | 47 | 2.39E-06 | 0.00027943 |
| BP | GO:0051052 | regulation of DNA metabolic process | 62 | 2.60E-06 | 0.00029088 |
| BP | GO:1990778 | protein localization to cell periphery | 47 | 2.65E-06 | 0.00029088 |
| BP | GO:0043484 | regulation of RNA splicing | 26 | 2.68E-06 | 0.00029088 |
| BP | GO:0016573 | histone acetylation | 31 | 2.75E-06 | 0.00029088 |
| BP | GO:0097485 | neuron projection guidance | 39 | 2.76E-06 | 0.00029088 |
| BP | GO:0051656 | establishment of organelle localization | 67 | 2.83E-06 | 0.000292905 |
| BP | GO:0097711 | ciliary basal body-plasma membrane docking | 23 | 2.90E-06 | 0.000294419 |
| BP | GO:0032886 | regulation of microtubule-based process | 37 | 3.80E-06 | 0.000379011 |
| BP | GO:0032386 | regulation of intracellular transport | 61 | 4.16E-06 | 0.00039164 |
| BP | GO:0006479 | protein methylation | 33 | 4.21E-06 | 0.00039164 |
| BP | GO:0008213 | protein alkylation | 33 | 4.21E-06 | 0.00039164 |
| BP | GO:0030705 | cytoskeleton-dependent intracellular transport | 31 | 4.24E-06 | 0.00039164 |
| BP | GO:1903313 | positive regulation of mRNA metabolic process | 19 | 4.31E-06 | 0.00039164 |
| BP | GO:0006403 | RNA localization | 38 | 4.37E-06 | 0.00039164 |
| BP | GO:0006333 | chromatin assembly or disassembly | 34 | 5.04E-06 | 0.000444222 |
| BP | GO:0006338 | chromatin remodeling | 31 | 5.60E-06 | 0.000485944 |
| BP | GO:1901796 | regulation of signal transduction by p53 class mediator | 34 | 5.72E-06 | 0.000488687 |
| BP | GO:0051261 | protein depolymerization | 22 | 7.10E-06 | 0.000596582 |
| BP | GO:0015931 | nucleobase-containing compound transport | 39 | 7.76E-06 | 0.000642079 |
| BP | GO:0071103 | DNA conformation change | 46 | 8.16E-06 | 0.000663612 |
| BP | GO:0061157 | mRNA destabilization | 11 | 8.26E-06 | 0.000663612 |
| BP | GO:0006397 | mRNA processing | 71 | 8.60E-06 | 0.000680511 |
| BP | GO:0098840 | protein transport along microtubule | 8 | 9.44E-06 | 0.000725428 |
| BP | GO:0099118 | microtubule-based protein transport | 8 | 9.44E-06 | 0.000725428 |
| BP | GO:0050808 | synapse organization | 59 | 1.05E-05 | 0.00079733 |
| BP | GO:0010975 | regulation of neuron projection development | 66 | 1.17E-05 | 0.000873857 |
| BP | GO:0031060 | regulation of histone methylation | 17 | 1.52E-05 | 0.001098904 |
| BP | GO:0006302 | double-strand break repair | 37 | 1.52E-05 | 0.001098904 |
| BP | GO:0048489 | synaptic vesicle transport | 26 | 1.55E-05 | 0.001098904 |
| BP | GO:0097480 | establishment of synaptic vesicle localization | 26 | 1.55E-05 | 0.001098904 |
| BP | GO:0051568 | histone H3-K4 methylation | 15 | 1.63E-05 | 0.001137737 |
| BP | GO:0051650 | establishment of vesicle localization | 42 | 1.68E-05 | 0.001156354 |
| BP | GO:0050779 | RNA destabilization | 11 | 1.79E-05 | 0.001209201 |
| BP | GO:0050769 | positive regulation of neurogenesis | 65 | 1.80E-05 | 0.001209201 |
| BP | GO:0034728 | nucleosome organization | 31 | 1.82E-05 | 0.001211089 |
| BP | GO:0010970 | transport along microtubule | 28 | 2.08E-05 | 0.001347721 |
| BP | GO:0099111 | microtubule-based transport | 28 | 2.08E-05 | 0.001347721 |
| BP | GO:0071824 | protein-DNA complex subunit organization | 40 | 2.40E-05 | 0.001533963 |
| BP | GO:0016079 | synaptic vesicle exocytosis | 22 | 2.42E-05 | 0.001533963 |
| BP | GO:0006836 | neurotransmitter transport | 40 | 2.65E-05 | 0.001657354 |
| BP | GO:0000724 | double-strand break repair via homologous recombination | 23 | 2.78E-05 | 0.001720001 |
| BP | GO:2001020 | regulation of response to DNA damage stimulus | 35 | 2.98E-05 | 0.001820313 |
| BP | GO:1902850 | microtubule cytoskeleton organization involved in mitosis | 24 | 3.11E-05 | 0.001870364 |
| BP | GO:0044091 | membrane biogenesis | 12 | 3.13E-05 | 0.001870364 |
| BP | GO:0008088 | axo-dendritic transport | 16 | 3.19E-05 | 0.001883078 |
| BP | GO:0000725 | recombinational repair | 23 | 3.25E-05 | 0.001899327 |
| BP | GO:0007052 | mitotic spindle organization | 21 | 3.41E-05 | 0.001974411 |
| BP | GO:0007051 | spindle organization | 28 | 3.52E-05 | 0.002012003 |
| BP | GO:0071709 | membrane assembly | 11 | 3.62E-05 | 0.002050079 |
| BP | GO:0000086 | G2/M transition of mitotic cell cycle | 39 | 4.45E-05 | 0.002414795 |
| BP | GO:0031503 | protein-containing complex localization | 39 | 4.45E-05 | 0.002414795 |
| BP | GO:0000731 | DNA synthesis involved in DNA repair | 14 | 4.48E-05 | 0.002414795 |
| BP | GO:0036297 | interstrand cross-link repair | 14 | 4.48E-05 | 0.002414795 |
| BP | GO:0061014 | positive regulation of mRNA catabolic process | 13 | 4.49E-05 | 0.002414795 |
| BP | GO:0007611 | learning or memory | 40 | 4.70E-05 | 0.002503535 |
| BP | GO:0043967 | histone H4 acetylation | 16 | 4.90E-05 | 0.002582937 |
| BP | GO:0010639 | negative regulation of organelle organization | 52 | 4.98E-05 | 0.0025996 |
| BP | GO:0044839 | cell cycle G2/M phase transition | 41 | 5.40E-05 | 0.00279247 |
| BP | GO:0031062 | positive regulation of histone methylation | 12 | 5.64E-05 | 0.00288785 |
| BP | GO:0051570 | regulation of histone H3-K9 methylation | 9 | 5.79E-05 | 0.002919542 |
| BP | GO:0007018 | microtubule-based movement | 37 | 5.81E-05 | 0.002919542 |
| BP | GO:0033044 | regulation of chromosome organization | 47 | 6.41E-05 | 0.003194262 |
| BP | GO:0098727 | maintenance of cell number | 29 | 6.63E-05 | 0.00327146 |
| BP | GO:0050804 | modulation of chemical synaptic transmission | 56 | 7.18E-05 | 0.00349836 |
| BP | GO:0000375 | RNA splicing, via transesterification reactions | 51 | 7.22E-05 | 0.00349836 |
| BP | GO:0010389 | regulation of G2/M transition of mitotic cell cycle | 32 | 7.32E-05 | 0.003515018 |
| BP | GO:0099177 | regulation of trans-synaptic signaling | 56 | 7.70E-05 | 0.003664514 |
| BP | GO:1902749 | regulation of cell cycle G2/M phase transition | 34 | 7.78E-05 | 0.003669403 |
| BP | GO:0006323 | DNA packaging | 33 | 7.97E-05 | 0.003730066 |
| BP | GO:0051028 | mRNA transport | 27 | 8.18E-05 | 0.003793464 |
| BP | GO:0032388 | positive regulation of intracellular transport | 34 | 8.60E-05 | 0.003953534 |
| BP | GO:0072659 | protein localization to plasma membrane | 38 | 8.93E-05 | 0.004058815 |
| BP | GO:0046785 | microtubule polymerization | 16 | 8.98E-05 | 0.004058815 |
| BP | GO:0033260 | nuclear DNA replication | 13 | 9.58E-05 | 0.004258183 |
| BP | GO:0045023 | G0 to G1 transition | 13 | 9.58E-05 | 0.004258183 |
| BP | GO:0070317 | negative regulation of G0 to G1 transition | 12 | 9.71E-05 | 0.004284148 |
| BP | GO:0051493 | regulation of cytoskeleton organization | 64 | 0.000101562 | 0.004442326 |
| BP | GO:0000377 | RNA splicing, via transesterification reactions with bulged adenosine as nucleophile | 50 | 0.00010429 | 0.004488661 |
| BP | GO:0000398 | mRNA splicing, via spliceosome | 50 | 0.00010429 | 0.004488661 |
| BP | GO:1903829 | positive regulation of cellular protein localization | 47 | 0.000110945 | 0.004737166 |
| BP | GO:0002483 | antigen processing and presentation of endogenous peptide antigen | 6 | 0.000113619 | 0.004813159 |
| BP | GO:0051053 | negative regulation of DNA metabolic process | 26 | 0.000115452 | 0.004852595 |
| BP | GO:0061647 | histone H3-K9 modification | 13 | 0.000121295 | 0.005058652 |
| BP | GO:0046928 | regulation of neurotransmitter secretion | 20 | 0.000125069 | 0.005175937 |
| BP | GO:0019827 | stem cell population maintenance | 28 | 0.000131424 | 0.005397401 |
| BP | GO:0050890 | cognition | 43 | 0.000144945 | 0.005907602 |
| BP | GO:0031110 | regulation of microtubule polymerization or depolymerization | 16 | 0.00015772 | 0.006349598 |
| BP | GO:0051168 | nuclear export | 30 | 0.000158762 | 0.006349598 |
| BP | GO:1902803 | regulation of synaptic vesicle transport | 17 | 0.00015933 | 0.006349598 |
| BP | GO:0051567 | histone H3-K9 methylation | 11 | 0.000161804 | 0.006400794 |
| BP | GO:0043981 | histone H4-K5 acetylation | 7 | 0.000173279 | 0.006755356 |
| BP | GO:0043982 | histone H4-K8 acetylation | 7 | 0.000173279 | 0.006755356 |
| BP | GO:0000018 | regulation of DNA recombination | 18 | 0.000184521 | 0.007095156 |
| BP | GO:0031497 | chromatin assembly | 27 | 0.000184632 | 0.007095156 |
| BP | GO:0140014 | mitotic nuclear division | 37 | 0.000191354 | 0.007301323 |
| BP | GO:0021537 | telencephalon development | 38 | 0.000197068 | 0.007466383 |
| BP | GO:0060271 | cilium assembly | 48 | 0.000200482 | 0.007542591 |
| BP | GO:0031111 | negative regulation of microtubule polymerization or depolymerization | 10 | 0.000202765 | 0.007575514 |
| BP | GO:0017157 | regulation of exocytosis | 31 | 0.000209002 | 0.007722338 |
| BP | GO:0035418 | protein localization to synapse | 14 | 0.000210076 | 0.007722338 |
| BP | GO:0099640 | axo-dendritic protein transport | 6 | 0.000211001 | 0.007722338 |
| BP | GO:2000300 | regulation of synaptic vesicle exocytosis | 16 | 0.000224728 | 0.008169171 |
| BP | GO:0045666 | positive regulation of neuron differentiation | 50 | 0.000227387 | 0.00821036 |
| BP | GO:0044782 | cilium organization | 49 | 0.000229362 | 0.008226439 |
| BP | GO:0098930 | axonal transport | 13 | 0.000235906 | 0.008405134 |
| BP | GO:0022604 | regulation of cell morphogenesis | 58 | 0.00024205 | 0.008567286 |
| BP | GO:0032201 | telomere maintenance via semi-conservative replication | 9 | 0.000243816 | 0.008573398 |
| BP | GO:0042177 | negative regulation of protein catabolic process | 23 | 0.000255366 | 0.008921217 |
| BP | GO:0070316 | regulation of G0 to G1 transition | 12 | 0.000257808 | 0.008948428 |
| BP | GO:0001505 | regulation of neurotransmitter levels | 46 | 0.000268565 | 0.009208313 |
| BP | GO:0048568 | embryonic organ development | 56 | 0.000268718 | 0.009208313 |
| BP | GO:1901214 | regulation of neuron death | 45 | 0.000289391 | 0.009853931 |
| BP | GO:0006275 | regulation of DNA replication | 20 | 0.00029543 | 0.009996312 |
| BP | GO:1990089 | response to nerve growth factor | 12 | 0.000322321 | 0.010838031 |
| BP | GO:0097484 | dendrite extension | 10 | 0.000352665 | 0.011784704 |
| BP | GO:0006334 | nucleosome assembly | 24 | 0.000366802 | 0.012036452 |
| BP | GO:0043523 | regulation of neuron apoptotic process | 33 | 0.00036691 | 0.012036452 |
| BP | GO:0065004 | protein-DNA complex assembly | 33 | 0.00036691 | 0.012036452 |
| BP | GO:0006297 | nucleotide-excision repair, DNA gap filling | 8 | 0.00038943 | 0.01262128 |
| BP | GO:0031114 | regulation of microtubule depolymerization | 8 | 0.00038943 | 0.01262128 |
| BP | GO:0007098 | centrosome cycle | 21 | 0.000399643 | 0.012809372 |
| BP | GO:0006301 | postreplication repair | 12 | 0.000399995 | 0.012809372 |
| BP | GO:1905477 | positive regulation of protein localization to membrane | 22 | 0.000407851 | 0.012983648 |
| BP | GO:0010976 | positive regulation of neuron projection development | 39 | 0.000446605 | 0.014133738 |
| BP | GO:0051588 | regulation of neurotransmitter transport | 23 | 0.000461286 | 0.014512975 |
| BP | GO:0090329 | regulation of DNA-dependent DNA replication | 12 | 0.000492895 | 0.01541731 |
| BP | GO:0031023 | microtubule organizing center organization | 22 | 0.000516819 | 0.016072175 |
| BP | GO:0017158 | regulation of calcium ion-dependent exocytosis | 18 | 0.000520854 | 0.016104554 |
| BP | GO:1903312 | negative regulation of mRNA metabolic process | 15 | 0.000526708 | 0.016192506 |
| BP | GO:0051098 | regulation of binding | 50 | 0.000532336 | 0.016201815 |
| BP | GO:0045739 | positive regulation of DNA repair | 14 | 0.000533034 | 0.016201815 |
| BP | GO:1905475 | regulation of protein localization to membrane | 29 | 0.000586745 | 0.017623562 |
| BP | GO:0019883 | antigen processing and presentation of endogenous antigen | 6 | 0.000591484 | 0.017623562 |
| BP | GO:0051169 | nuclear transport | 43 | 0.000591486 | 0.017623562 |
| BP | GO:0043044 | ATP-dependent chromatin remodeling | 16 | 0.000592912 | 0.017623562 |
| BP | GO:0016358 | dendrite development | 33 | 0.000612743 | 0.018112943 |
| BP | GO:0008089 | anterograde axonal transport | 11 | 0.000679976 | 0.019874484 |
| BP | GO:1990090 | cellular response to nerve growth factor stimulus | 11 | 0.000679976 | 0.019874484 |
| BP | GO:1901216 | positive regulation of neuron death | 18 | 0.000683416 | 0.019874484 |
| BP | GO:0045814 | negative regulation of gene expression, epigenetic | 19 | 0.000709437 | 0.020520275 |
| BP | GO:2001251 | negative regulation of chromosome organization | 22 | 0.000727597 | 0.020876676 |
| BP | GO:0071897 | DNA biosynthetic process | 30 | 0.00072952 | 0.020876676 |
| BP | GO:0036465 | synaptic vesicle recycling | 12 | 0.000733722 | 0.020885856 |
| BP | GO:0042023 | DNA endoreduplication | 5 | 0.000745029 | 0.021096093 |
| BP | GO:0007612 | learning | 24 | 0.000781138 | 0.022002744 |
| BP | GO:2001021 | negative regulation of response to DNA damage stimulus | 16 | 0.000796922 | 0.022330419 |
| BP | GO:0043984 | histone H4-K16 acetylation | 7 | 0.000868958 | 0.024222758 |
| BP | GO:0006913 | nucleocytoplasmic transport | 42 | 0.000897981 | 0.024902776 |
| BP | GO:0007158 | neuron cell-cell adhesion | 6 | 0.000915729 | 0.025008243 |
| BP | GO:0043584 | nose development | 6 | 0.000915729 | 0.025008243 |
| BP | GO:0061158 | 3'-UTR-mediated mRNA destabilization | 6 | 0.000915729 | 0.025008243 |
| BP | GO:0001764 | neuron migration | 25 | 0.000923855 | 0.025102725 |
| BP | GO:0031058 | positive regulation of histone modification | 17 | 0.000973209 | 0.026310866 |
| BP | GO:0040018 | positive regulation of multicellular organism growth | 9 | 0.00099253 | 0.026587226 |
| BP | GO:0043414 | macromolecule methylation | 39 | 0.000993315 | 0.026587226 |
| BP | GO:0006282 | regulation of DNA repair | 20 | 0.001031531 | 0.027473442 |
| BP | GO:0090307 | mitotic spindle assembly | 12 | 0.001065804 | 0.028241564 |
| BP | GO:0051100 | negative regulation of binding | 26 | 0.00107087 | 0.028241564 |
| BP | GO:0010769 | regulation of cell morphogenesis involved in differentiation | 37 | 0.001159253 | 0.030423335 |
| BP | GO:0043524 | negative regulation of neuron apoptotic process | 24 | 0.001166122 | 0.030455025 |
| BP | GO:0006611 | protein export from nucleus | 26 | 0.001174179 | 0.030517313 |
| BP | GO:0007026 | negative regulation of microtubule depolymerization | 7 | 0.001209283 | 0.030980675 |
| BP | GO:0099563 | modification of synaptic structure | 7 | 0.001209283 | 0.030980675 |
| BP | GO:2000104 | negative regulation of DNA-dependent DNA replication | 7 | 0.001209283 | 0.030980675 |
| BP | GO:0051225 | spindle assembly | 17 | 0.001263331 | 0.032211938 |
| BP | GO:0051574 | positive regulation of histone H3-K9 methylation | 5 | 0.001271204 | 0.032259811 |
| BP | GO:0042752 | regulation of circadian rhythm | 20 | 0.001293175 | 0.032663302 |
| BP | GO:1902751 | positive regulation of cell cycle G2/M phase transition | 8 | 0.001300393 | 0.032692134 |
| BP | GO:0060968 | regulation of gene silencing | 23 | 0.001334943 | 0.033404627 |
| BP | GO:0060627 | regulation of vesicle-mediated transport | 60 | 0.001342232 | 0.033431512 |
| BP | GO:0008090 | retrograde axonal transport | 6 | 0.0013612 | 0.033747717 |
| BP | GO:0048813 | dendrite morphogenesis | 22 | 0.001379677 | 0.034048907 |
| BP | GO:0019985 | translesion synthesis | 10 | 0.001430802 | 0.035149381 |
| BP | GO:0070997 | neuron death | 46 | 0.001455458 | 0.035592552 |
| BP | GO:0000819 | sister chromatid segregation | 25 | 0.00148085 | 0.036049659 |
| BP | GO:0031113 | regulation of microtubule polymerization | 11 | 0.001506498 | 0.036508826 |
| BP | GO:0035264 | multicellular organism growth | 24 | 0.001553231 | 0.037472573 |
| BP | GO:2001022 | positive regulation of response to DNA damage stimulus | 17 | 0.001623508 | 0.038993179 |
| BP | GO:0010842 | retina layer formation | 7 | 0.001645687 | 0.039176097 |
| BP | GO:0032594 | protein transport within lipid bilayer | 7 | 0.001645687 | 0.039176097 |
| BP | GO:0032200 | telomere organization | 26 | 0.001678281 | 0.039670197 |
| BP | GO:0051402 | neuron apoptotic process | 34 | 0.00168119 | 0.039670197 |
| BP | GO:0090316 | positive regulation of intracellular protein transport | 24 | 0.001704642 | 0.040047915 |
| BP | GO:0007059 | chromosome segregation | 38 | 0.001726847 | 0.0403932 |
| BP | GO:0010569 | regulation of double-strand break repair via homologous recombination | 10 | 0.001751443 | 0.040615358 |
| BP | GO:0043489 | RNA stabilization | 10 | 0.001751443 | 0.040615358 |
| BP | GO:1902903 | regulation of supramolecular fiber organization | 42 | 0.001812605 | 0.04185329 |
| BP | GO:1901990 | regulation of mitotic cell cycle phase transition | 54 | 0.001853464 | 0.042613841 |
| BP | GO:0001701 | in utero embryonic development | 45 | 0.001890102 | 0.043271278 |
| BP | GO:0051668 | localization within membrane | 21 | 0.001930072 | 0.043999093 |
| BP | GO:0097329 | response to antimetabolite | 6 | 0.001954669 | 0.0443718 |
| BP | GO:0045622 | regulation of T-helper cell differentiation | 9 | 0.001984736 | 0.044686459 |
| BP | GO:0048167 | regulation of synaptic plasticity | 26 | 0.001993448 | 0.044686459 |
| BP | GO:2001252 | positive regulation of chromosome organization | 26 | 0.001993448 | 0.044686459 |
| BP | GO:0016082 | synaptic vesicle priming | 5 | 0.002028493 | 0.044910664 |
| BP | GO:0072520 | seminiferous tubule development | 5 | 0.002028493 | 0.044910664 |
| BP | GO:0072710 | response to hydroxyurea | 5 | 0.002028493 | 0.044910664 |
| BP | GO:1902369 | negative regulation of RNA catabolic process | 11 | 0.002156521 | 0.047438181 |
| BP | GO:0097106 | postsynaptic density organization | 8 | 0.002160289 | 0.047438181 |
| BP | GO:0009895 | negative regulation of catabolic process | 39 | 0.002192775 | 0.047816656 |
| BP | GO:0097150 | neuronal stem cell population maintenance | 7 | 0.0021953 | 0.047816656 |
| BP | GO:1903305 | regulation of regulated secretory pathway | 22 | 0.002255864 | 0.048937689 |
| BP | GO:0099565 | chemical synaptic transmission, postsynaptic | 18 | 0.002306131 | 0.049827238 |
| CC | GO:0000785 | chromatin | 96 | 1.17E-15 | 3.68E-13 |
| CC | GO:0044454 | nuclear chromosome part | 92 | 1.19E-15 | 3.68E-13 |
| CC | GO:0000790 | nuclear chromatin | 66 | 2.37E-13 | 4.87E-11 |
| CC | GO:0098687 | chromosomal region | 64 | 1.18E-10 | 1.82E-08 |
| CC | GO:0044450 | microtubule organizing center part | 39 | 2.21E-09 | 2.73E-07 |
| CC | GO:0033267 | axon part | 62 | 6.33E-09 | 5.65E-07 |
| CC | GO:0098793 | presynapse | 72 | 6.41E-09 | 5.65E-07 |
| CC | GO:0005813 | centrosome | 75 | 1.53E-08 | 1.18E-06 |
| CC | GO:0000775 | chromosome, centromeric region | 39 | 2.72E-08 | 1.86E-06 |
| CC | GO:0035770 | ribonucleoprotein granule | 39 | 4.30E-08 | 2.66E-06 |
| CC | GO:0036464 | cytoplasmic ribonucleoprotein granule | 37 | 6.61E-08 | 3.71E-06 |
| CC | GO:0048786 | presynaptic active zone | 19 | 1.41E-07 | 7.27E-06 |
| CC | GO:0000793 | condensed chromosome | 39 | 2.10E-07 | 9.98E-06 |
| CC | GO:0005635 | nuclear envelope | 65 | 3.43E-07 | 1.51E-05 |
| CC | GO:0005819 | spindle | 54 | 3.87E-07 | 1.59E-05 |
| CC | GO:0005814 | centriole | 28 | 5.57E-07 | 2.15E-05 |
| CC | GO:0070603 | SWI/SNF superfamily-type complex | 19 | 1.23E-06 | 4.23E-05 |
| CC | GO:0150034 | distal axon | 45 | 1.29E-06 | 4.23E-05 |
| CC | GO:0043025 | neuronal cell body | 67 | 1.30E-06 | 4.23E-05 |
| CC | GO:0005874 | microtubule | 56 | 1.43E-06 | 4.41E-05 |
| CC | GO:0000932 | P-body | 18 | 1.64E-06 | 4.82E-05 |
| CC | GO:0034399 | nuclear periphery | 27 | 2.59E-06 | 7.24E-05 |
| CC | GO:0035097 | histone methyltransferase complex | 18 | 2.70E-06 | 7.24E-05 |
| CC | GO:1904949 | ATPase complex | 22 | 3.26E-06 | 8.25E-05 |
| CC | GO:0016363 | nuclear matrix | 24 | 3.34E-06 | 8.25E-05 |
| CC | GO:0120111 | neuron projection cytoplasm | 20 | 4.36E-06 | 0.000103414 |
| CC | GO:0034708 | methyltransferase complex | 21 | 5.61E-06 | 0.000128254 |
| CC | GO:0000792 | heterochromatin | 19 | 6.10E-06 | 0.000134352 |
| CC | GO:0005667 | transcription factor complex | 44 | 6.95E-06 | 0.000147805 |
| CC | GO:0016607 | nuclear speck | 57 | 7.78E-06 | 0.000159976 |
| CC | GO:0045335 | phagocytic vesicle | 26 | 1.10E-05 | 0.000218879 |
| CC | GO:0016605 | PML body | 22 | 1.16E-05 | 0.000224093 |
| CC | GO:0000922 | spindle pole | 29 | 1.26E-05 | 0.000235612 |
| CC | GO:0098984 | neuron to neuron synapse | 48 | 1.73E-05 | 0.000314585 |
| CC | GO:0000779 | condensed chromosome, centromeric region | 23 | 2.13E-05 | 0.00037567 |
| CC | GO:0044306 | neuron projection terminus | 24 | 3.09E-05 | 0.000529486 |
| CC | GO:0014069 | postsynaptic density | 44 | 4.25E-05 | 0.000708223 |
| CC | GO:0071565 | nBAF complex | 7 | 4.78E-05 | 0.000775384 |
| CC | GO:0043679 | axon terminus | 21 | 5.23E-05 | 0.00082678 |
| CC | GO:0090575 | RNA polymerase II transcription factor complex | 24 | 5.39E-05 | 0.000831329 |
| CC | GO:0000781 | chromosome, telomeric region | 28 | 5.58E-05 | 0.000840021 |
| CC | GO:0032279 | asymmetric synapse | 44 | 5.92E-05 | 0.000851944 |
| CC | GO:0097060 | synaptic membrane | 55 | 5.94E-05 | 0.000851944 |
| CC | GO:0042734 | presynaptic membrane | 26 | 6.35E-05 | 0.000891025 |
| CC | GO:0030670 | phagocytic vesicle membrane | 17 | 7.17E-05 | 0.000983471 |
| CC | GO:1904115 | axon cytoplasm | 14 | 7.61E-05 | 0.001000157 |
| CC | GO:0098978 | glutamatergic synapse | 50 | 7.62E-05 | 0.001000157 |
| CC | GO:0031519 | PcG protein complex | 12 | 8.86E-05 | 0.00113841 |
| CC | GO:0031010 | ISWI-type complex | 6 | 9.25E-05 | 0.001165022 |
| CC | GO:0005721 | pericentric heterochromatin | 8 | 9.72E-05 | 0.001199741 |
| CC | GO:0000776 | kinetochore | 24 | 0.000132812 | 0.001602033 |
| CC | GO:0000777 | condensed chromosome kinetochore | 20 | 0.000137517 | 0.001602033 |
| CC | GO:0035098 | ESC/E(Z) complex | 7 | 0.000137614 | 0.001602033 |
| CC | GO:0044798 | nuclear transcription factor complex | 25 | 0.000148536 | 0.001697164 |
| CC | GO:0099572 | postsynaptic specialization | 45 | 0.000160518 | 0.001800721 |
| CC | GO:0005643 | nuclear pore | 14 | 0.000212188 | 0.002296839 |
| CC | GO:0098839 | postsynaptic density membrane | 14 | 0.000212188 | 0.002296839 |
| CC | GO:0060076 | excitatory synapse | 12 | 0.000229806 | 0.002444658 |
| CC | GO:0005657 | replication fork | 13 | 0.000303762 | 0.00317663 |
| CC | GO:0032838 | plasma membrane bounded cell projection cytoplasm | 29 | 0.000324214 | 0.003334 |
| CC | GO:0008021 | synaptic vesicle | 26 | 0.000345965 | 0.00349935 |
| CC | GO:0031965 | nuclear membrane | 40 | 0.000364428 | 0.003626642 |
| CC | GO:0099634 | postsynaptic specialization membrane | 17 | 0.000490249 | 0.004801324 |
| CC | GO:0043195 | terminal bouton | 10 | 0.000556721 | 0.005367136 |
| CC | GO:0099568 | cytoplasmic region | 52 | 0.000574573 | 0.005410894 |
| CC | GO:0000123 | histone acetyltransferase complex | 15 | 0.000578799 | 0.005410894 |
| CC | GO:0044304 | main axon | 14 | 0.000614322 | 0.005468933 |
| CC | GO:0090734 | site of DNA damage | 14 | 0.000614322 | 0.005468933 |
| CC | GO:0031248 | protein acetyltransferase complex | 16 | 0.000620462 | 0.005468933 |
| CC | GO:1902493 | acetyltransferase complex | 16 | 0.000620462 | 0.005468933 |
| CC | GO:0030496 | midbody | 27 | 0.000646427 | 0.005617539 |
| CC | GO:0000784 | nuclear chromosome, telomeric region | 21 | 0.000718568 | 0.006157728 |
| CC | GO:0070382 | exocytic vesicle | 26 | 0.000911182 | 0.007701364 |
| CC | GO:0099055 | integral component of postsynaptic membrane | 20 | 0.00092607 | 0.007721425 |
| CC | GO:0005720 | nuclear heterochromatin | 9 | 0.000974338 | 0.008015558 |
| CC | GO:0045211 | postsynaptic membrane | 40 | 0.001250873 | 0.010155117 |
| CC | GO:0010494 | cytoplasmic stress granule | 12 | 0.001309663 | 0.010494311 |
| CC | GO:0033116 | endoplasmic reticulum-Golgi intermediate compartment membrane | 14 | 0.001328214 | 0.010506516 |
| CC | GO:0099061 | integral component of postsynaptic density membrane | 11 | 0.001346827 | 0.010518891 |
| CC | GO:0099060 | integral component of postsynaptic specialization membrane | 14 | 0.001532904 | 0.011822519 |
| CC | GO:0098936 | intrinsic component of postsynaptic membrane | 20 | 0.001588899 | 0.011822851 |
| CC | GO:0030672 | synaptic vesicle membrane | 16 | 0.001590432 | 0.011822851 |
| CC | GO:0099501 | exocytic vesicle membrane | 16 | 0.001590432 | 0.011822851 |
| CC | GO:0008278 | cohesin complex | 5 | 0.001721589 | 0.012645483 |
| CC | GO:0016234 | inclusion body | 15 | 0.002045918 | 0.014850955 |
| CC | GO:0032839 | dendrite cytoplasm | 8 | 0.002169348 | 0.015563812 |
| CC | GO:0032433 | filopodium tip | 6 | 0.002261131 | 0.01589376 |
| CC | GO:0099146 | intrinsic component of postsynaptic density membrane | 11 | 0.002266857 | 0.01589376 |
| CC | GO:0098948 | intrinsic component of postsynaptic specialization membrane | 14 | 0.002310374 | 0.016016863 |
| CC | GO:0030133 | transport vesicle | 44 | 0.002417227 | 0.016571437 |
| CC | GO:0043240 | Fanconi anaemia nuclear complex | 5 | 0.002611361 | 0.017705598 |
| CC | GO:0043204 | perikaryon | 20 | 0.003162791 | 0.021211327 |
| CC | GO:0016581 | NuRD complex | 5 | 0.003792335 | 0.024593654 |
| CC | GO:0090545 | CHD-type complex | 5 | 0.003792335 | 0.024593654 |
| CC | GO:0005881 | cytoplasmic microtubule | 12 | 0.003804624 | 0.024593654 |
| CC | GO:0017053 | transcriptional repressor complex | 14 | 0.003826565 | 0.024593654 |
| CC | GO:0000242 | pericentriolar material | 6 | 0.004100527 | 0.025982511 |
| CC | GO:0030426 | growth cone | 24 | 0.004126882 | 0.025982511 |
| CC | GO:0043596 | nuclear replication fork | 7 | 0.004831527 | 0.030111637 |
| CC | GO:0072686 | mitotic spindle | 16 | 0.004947756 | 0.030527655 |
| CC | GO:0030427 | site of polarized growth | 24 | 0.00518646 | 0.031555241 |
| CC | GO:0030673 | axolemma | 5 | 0.005311581 | 0.031555241 |
| CC | GO:0045120 | pronucleus | 5 | 0.005311581 | 0.031555241 |
| CC | GO:0030658 | transport vesicle membrane | 26 | 0.005318874 | 0.031555241 |
| CC | GO:0099699 | integral component of synaptic membrane | 22 | 0.005783202 | 0.033983198 |
| CC | GO:0019898 | extrinsic component of membrane | 27 | 0.005920117 | 0.034459549 |
| CC | GO:0098685 | Schaffer collateral - CA1 synapse | 14 | 0.006067944 | 0.034989919 |
| CC | GO:0000794 | condensed nuclear chromosome | 13 | 0.00705567 | 0.040308782 |
| CC | GO:0035861 | site of double-strand break | 10 | 0.007214088 | 0.040692541 |
| CC | GO:0043198 | dendritic shaft | 8 | 0.007254748 | 0.040692541 |
| CC | GO:0042555 | MCM complex | 4 | 0.009030047 | 0.049745885 |
| CC | GO:0048188 | Set1C/COMPASS complex | 4 | 0.009030047 | 0.049745885 |
| MF | GO:0003682 | chromatin binding | 91 | 7.78E-15 | 6.48E-12 |
| MF | GO:0000987 | proximal promoter sequence-specific DNA binding | 76 | 3.94E-08 | 1.64E-05 |
| MF | GO:0003730 | mRNA 3'-UTR binding | 20 | 8.28E-07 | 0.000209998 |
| MF | GO:0000978 | RNA polymerase II proximal promoter sequence-specific DNA binding | 69 | 1.17E-06 | 0.000209998 |
| MF | GO:0042393 | histone binding | 36 | 1.28E-06 | 0.000209998 |
| MF | GO:0001085 | RNA polymerase II transcription factor binding | 27 | 1.51E-06 | 0.000209998 |
| MF | GO:0017091 | AU-rich element binding | 11 | 3.65E-06 | 0.000380354 |
| MF | GO:0035925 | mRNA 3'-UTR AU-rich region binding | 11 | 3.65E-06 | 0.000380354 |
| MF | GO:0015631 | tubulin binding | 44 | 1.07E-05 | 0.000986136 |
| MF | GO:0048306 | calcium-dependent protein binding | 16 | 3.07E-05 | 0.002561082 |
| MF | GO:0042826 | histone deacetylase binding | 21 | 0.000213688 | 0.016181991 |
| MF | GO:0061980 | regulatory RNA binding | 11 | 0.000257718 | 0.017889923 |
| MF | GO:0003712 | transcription coregulator activity | 67 | 0.000318339 | 0.018827894 |
| MF | GO:0008017 | microtubule binding | 29 | 0.000318701 | 0.018827894 |
| MF | GO:0004386 | helicase activity | 24 | 0.000350032 | 0.018827894 |
| MF | GO:0030507 | spectrin binding | 9 | 0.000362563 | 0.018827894 |
| MF | GO:0140097 | catalytic activity, acting on DNA | 27 | 0.000384243 | 0.018827894 |
| MF | GO:0048156 | tau protein binding | 12 | 0.00041935 | 0.01940657 |
| MF | GO:0018024 | histone-lysine N-methyltransferase activity | 11 | 0.001055304 | 0.046266729 |
| MF | GO:0035198 | miRNA binding | 9 | 0.00112627 | 0.046909126 |

Note: GO, Gene Ontology.
